# Comparative effectiveness of traditional Chinese non-pharmacological therapies for chemotherapy-related symptoms in cancer patients: a systematic review and network meta-analysis

**DOI:** 10.1007/s00520-026-10990-7

**Published:** 2026-07-27

**Authors:** Xinzheng Zhang, Junyong Song, Huiyuan Liu, Yulong Wei

**Affiliations:** 1 Department of Acupuncture and Massage, Beijing University of Chinese Medicine, Beijing, 100029 China; 2 The Second Clinical Medical College of Beijing University of Chinese Medicine, Beijing, 100078 China; 3 Xiyuan Hospital of Chinese Academy of Traditional Chinese Medicine, Beijing, 100091 China

**Keywords:** Cancer, Traditional Chinese medicine, Non-pharmacological therapy, Chemotherapy-related symptoms, Network meta-analysis

## Abstract

**Background:**

Chemotherapy-related fatigue, sleep disturbance, and psychological distress significantly impair quality of life (QoL) in cancer patients. Traditional Chinese medicine (TCM) non-pharmacological therapies are increasingly used as supportive care, yet their comparative effectiveness remains unclear. This study aimed to evaluate and compare five TCM-based interventions for improving QoL and emotional well-being in chemotherapy patients.

**Methods:**

A systematic review and network meta-analysis was conducted, including randomized controlled trials (RCTs) published through April 2025. Five TCM-based interventions (acupuncture, auricular therapy, manual acupoint therapy, mind–body exercise therapy, and moxibustion) were evaluated. Primary outcomes included QoL, sleep quality, fatigue, general mood, anxiety, and depression. Effect sizes were calculated as standardized mean differences (SMDs) with 95% confidence intervals (CIs). Treatment ranking was determined using surface under the cumulative ranking curve (SUCRA).

**Results:**

Thirty-five RCTs involving 2025 chemotherapy patients were included. Manual acupoint therapy ranked highest for improving QoL (SUCRA 63.4%; SMD vs. control, 2.29; 95% CI, 0.95 to 3.63) and sleep quality (SUCRA 91.9%; SMD vs. auricular therapy, −9.92; 95% CI, −14.83 to −5.01). Mind–body exercise therapy was most effective for enhancing mood (SUCRA 68.0%; SMD vs. control, 0.72; 95% CI, 0.20 to 1.24) and reducing depression (SUCRA 70.8%; SMD vs. control, −0.92; 95% CI, −1.79 to −0.05). Auricular therapy showed the greatest benefit for fatigue (SUCRA 100%; SMD vs. control, −8.13; 95% CI, −10.15 to −6.11) and anxiety (SUCRA 92.9%; SMD vs. control, −1.53; 95% CI, −2.48 to −0.58).

**Conclusion:**

TCM non-pharmacological therapies demonstrate distinct, symptom-specific efficacy in cancer patients undergoing chemotherapy. Manual acupoint therapy is most effective for QoL and sleep, mind–body exercise therapy for mood and depression, and auricular therapy for fatigue and anxiety. These findings support individualized integration of TCM interventions into supportive cancer care.

**Supplementary information:**

The online version contains supplementary material available at 10.1007/s00520-026-10990-7.

## Introduction

Cancer remains one of the leading causes of morbidity and mortality worldwide. As treatment paradigms evolve, chemotherapy continues to serve as a cornerstone in the management of various malignancies [1]. However, despite its clinical efficacy, chemotherapy is frequently associated with a wide spectrum of physical and psychological side effects that significantly compromise patients’ health-related quality of life (HRQoL) [2]. Chemotherapy-induced fatigue, insomnia, and gastrointestinal disturbances are often accompanied by emotional challenges, including heightened levels of anxiety and depression. These psychological comorbidities are not merely transient but can persist throughout survivorship, exerting a substantial toll on patients’ social functioning, treatment adherence, and overall prognosis [3, 4]. With the global burden of cancer expected to surpass 30 million new cases by 2040, the psychosocial sequelae of chemotherapy pose a growing public health concern, with implications for healthcare utilization, workforce participation, and long-term caregiving needs [5].

To mitigate these burdens, a range of interventions has been proposed. Conventional approaches, such as pharmacotherapy and cognitive behavioral therapy (CBT), have demonstrated benefits in managing psychological distress and improving QoL among cancer patients [6]. However, pharmacological treatments often carry the risk of adverse effects, including sedation, drug interactions, and dependence—particularly in populations already managing polypharmacy [7]. Similarly, while CBT is efficacious, access remains limited by cost, availability of trained providers, and persistent stigma surrounding mental health care [8]. These limitations have prompted increasing interest in integrative, patient-centered approaches. Traditional Chinese medicine (TCM), with its holistic philosophy and individualized treatment strategies, has gained attention as a complementary framework. Although Chinese herbal formulations have been widely used, concerns regarding herb–drug interactions and regulatory variability have restricted their broader adoption, highlighting the need to explore the potential of non-pharmacological TCM modalities [9].

A growing body of clinical research has investigated the role of TCM-based non-pharmacological therapies—such as acupuncture, moxibustion, auricular therapy, Tuina massage, Tai Chi, and Qigong—in alleviating psychological distress and enhancing QoL in cancer patients undergoing chemotherapy [10]. These interventions are rooted in the regulation of “Qi,” stimulation of specific acupoints, and balancing of the autonomic nervous system, offering a multifaceted approach to symptom management. Several randomized controlled trials (RCTs) and conventional meta-analyses have demonstrated the efficacy of individual TCM modalities for specific outcomes, such as fatigue reduction or sleep improvement [11–13]. However, the relative comparative effectiveness of different TCM non-pharmacological interventions remains unclear. Current evidence synthesis is limited by the lack of head-to-head comparisons, small sample sizes, and methodological heterogeneity across trials, hindering evidence-based clinical decision-making.

Network meta-analysis (NMA) offers a powerful solution by integrating direct and indirect evidence to estimate the comparative efficacy of multiple interventions within a single analytic framework. By enabling the ranking of interventions according to their relative performance, NMA provides critical insights for clinicians and policymakers seeking to optimize supportive cancer care [14]. Therefore, this systematic review and network meta-analysis aimed to evaluate and compare the effects of various TCM non-pharmacological therapies on quality of life, fatigue, sleep quality, and emotional well-being in cancer patients undergoing chemotherapy. Our study seeks to inform evidence-based recommendations, guide integrative oncology practice, and promote the development of personalized supportive care strategies tailored to patients’ therapeutic preferences and cultural contexts.

## Methods

This systematic review and network meta-analysis was conducted and reported in accordance with the Preferred Reporting Items for Systematic Reviews and Meta-Analyses (PRISMA) guidelines (2020) and the PRISMA extension statement for systematic reviews incorporating network meta-analyses of healthcare interventions [15, 16]. This review was prospectively registered with PROSPERO (Registration number: CRD420251069168, registered on 29 June 2025). Given the nature of this study, ethical approval and informed consent were not required.

### Data sources and search strategy

A comprehensive literature search was conducted using PubMed, Medline, Embase, PsycINFO, Cochrane Central Register of Controlled Trials (CENTRAL), and Web of Science databases from their respective inception dates to April 30, 2025. To capture TCM trials published in Chinese, we additionally searched CNKI, WanFang Data, the VIP Chinese Science and Technology Periodical Database, and SinoMed from inception to the same end date. The search terms included combinations of keywords related to “cancer,” “chemotherapy,” traditional Chinese medicine (TCM) non-pharmacological interventions (e.g., “acupuncture,” “moxibustion,” “Tai Chi,” “massage”), and outcomes such as “quality of life,” “mood,” “depression,” and “anxiety.” The complete search strategy, including specific search terms and Boolean operators, is provided in Supplementary File 1. No language restrictions were applied. Additionally, reference lists of included studies and bibliographies of systematic reviews published in the past 5 years were manually screened to identify potentially relevant studies. Two independent reviewers screened titles, abstracts, and full texts, resolving discrepancies through discussion or consultation with a third reviewer.

### Study selection

Studies were included if they met the following criteria: (1) population: adults receiving chemotherapy for cancer; (2) intervention: TCM non-pharmacological treatments, such as acupuncture, massage, moxibustion, and Tai Chi; (3) comparison: control groups comprising routine care or different TCM non-pharmacological interventions for head-to-head network comparisons; (4) outcomes: measures assessing quality of life, sleep quality, fatigue, general mood, anxiety, and depression among cancer patients; (5) study design: randomized controlled trials (RCTs); (6) language: published in English.

Exclusion criteria were as follows: (1) studies involving cancer patients not receiving chemotherapy or pediatric populations; (2) interventions combined with Western pharmacotherapy or Chinese herbal medicine; (3) studies lacking a clear description of interventions; (4) studies not reporting means and standard deviations (SD) for outcomes and whose authors did not respond to data requests; (5) study protocols, animal experiments, or conference abstracts. Two reviewers independently assessed eligibility by reviewing titles, abstracts, and full texts according to predefined inclusion and exclusion criteria.

### Data extraction

Eligible studies were managed using EndNote X9 software to prevent duplication. Two independent reviewers extracted relevant data, including bibliographic details (authors, title, country, publication year), participant characteristics (sample size, age, sex, cancer types), intervention details (treatment protocols for both intervention and control groups, duration of treatment), and outcome measures (Supplementary File 2). A standardized extraction form was used, and the full template is provided in Supplementary File 2. Missing means and SDs were estimated following recommendations from the Cochrane Handbook [17]. For studies with missing outcome data, corresponding authors were contacted by email on up to four occasions over a 6-week period; when unavailable after contact, missing SDs were imputed using Cochrane-recommended approaches. The missing data status and handling for each study are summarized in Supplementary File 2. In cases of incomplete data, the corresponding authors were contacted at least four times over 6 weeks. If no response was received after these attempts, the study was excluded from quantitative synthesis.

### Risk of bias assessment

Two reviewers independently assessed the risk of bias in each included study using the revised Cochrane risk-of-bias tool (RoB 2), focusing on domains such as randomization processes, deviations from intended interventions, missing outcome data, measurement of outcomes, and selective reporting [18]. Any disagreements were resolved by consultation with a third reviewer.

### Data coding

Interventions in the included studies were categorized and coded into five distinct groups based on their underlying therapeutic mechanisms and modality characteristics: (1) acupuncture, including manual and electro-acupuncture; (2) auricular therapy, such as auricular acupuncture and auricular acupressure; (3) manual acupoint therapy, comprising Tuina massage and body acupressure techniques; (4) mind–body exercise therapy, including Tai Chi and Qigong, which integrate physical movement, breath control, and meditative focus; and (5) moxibustion, involving the application of heat to acupuncture points using burning moxa. Routine care or wait-list control groups were uniformly coded as “CON.”

To enhance transparency and reproducibility, each intervention node was defined using a structured, guideline-informed framework, with representative examples provided for illustrative purposes. Detailed definitions, examples, and supporting references are presented in Supplementary File 3. Because intervention parameters varied across trials, these characteristics are presented as representative patterns rather than mandatory criteria.

### Outcome measures

The primary outcomes assessed in this study encompassed multiple dimensions of health-related quality of life and psychological well-being: quality of life, sleep quality, fatigue, general mood, anxiety, and depression. Quality of life was primarily evaluated using validated instruments such as the European Organisation for Research and Treatment of Cancer Quality of Life Questionnaire (EORTC QLQ-C30) and the Functional Assessment of Cancer Therapy-General (FACT-G). Sleep quality was assessed using the Pittsburgh Sleep Quality Index (PSQI), while fatigue was measured by tools like the Brief Fatigue Inventory (BFI) and the Multidimensional Fatigue Symptom Inventory-Short Form (MFSI-SF). Anxiety and depression were commonly evaluated using the Hospital Anxiety and Depression Scale (HADS) and the Self-Rating Depression and Anxiety Scales (SDS/SAS).

General mood was defined as overall emotional functioning, typically assessed using the EORTC QLQ-C30 Emotional Functioning domain (items 24–27), scored 0–100 with higher scores indicating better mood. In studies not using the EORTC QLQ-C30, equivalent emotional subscales from other validated instruments were used. General mood was included separately as it captures broader emotional well-being not fully reflected by anxiety or depression scales.

### Data analysis

All statistical analyses were conducted using Stata software (version 17.0; StataCorp LLC, Texas, USA). Network meta-analysis was applied to simultaneously compare the relative effectiveness of different TCM non-pharmacological interventions. A network diagram was constructed to visualize the geometry of treatment comparisons and to assess network connectivity. Due to clinical and methodological heterogeneity, a random-effects model was adopted using a frequentist framework. A frequentist network meta-analysis approach was chosen to ensure consistency with prior comparative effectiveness research in supportive and integrative oncology and to facilitate transparent estimation of relative treatment effects and ranking probabilities without reliance on prior assumptions.

Effect sizes were expressed as standardized mean differences (SMDs) with 95% confidence intervals (CIs), enabling comparison across different measurement tools. As included trials used heterogeneous instruments for the same construct, outcomes were synthesized on a standardized scale. To ensure interpretability, all scales were coded so that a consistent direction of benefit was applied before pooling. Specifically, negative values indicate improvement for symptom severity outcomes (sleep, fatigue, anxiety, and depression), whereas positive values indicate improvement for functioning outcomes (quality of life and emotional functioning). Heterogeneity was assessed with the I2 statistic, with thresholds of 25%, 50%, and 75% representing low, moderate, and high heterogeneity, respectively.

Global inconsistency within each network was first assessed using the design-by-treatment interaction model. For outcomes in which statistically significant global inconsistency was detected, local inconsistency between direct and indirect evidence was further examined using a node-splitting approach. The results of global inconsistency tests are presented in Supplementary File 4, and detailed node-splitting analyses are reported in Supplementary File 5. Ranking probabilities of each treatment were calculated based on the surface under the cumulative ranking (SUCRA) curve, where higher SUCRA values indicate greater treatment efficacy.

To assess clinical relevance, we linked effect sizes to minimal clinically important differences (MCIDs): EORTC QLQ-C30 for QoL (10 points), PSQI for sleep (3 points), BFI for fatigue (1.3 points), and HADS for anxiety/depression (3 points). For example, the SMD of −1.92 for sleep quality corresponds to a reduction of 4.2 points on the PSQI, exceeding the MCID of 3 points, suggesting clinical benefit.

Bayesian sensitivity analyses were performed to verify robustness, utilizing Markov Chain Monte Carlo simulations via the “network” and “mvmeta” packages in Stata. To assess potential publication bias, comparison-adjusted funnel plots were generated, and Egger’s tests were conducted, with *p*-values < 0.05 suggesting bias [19]. Predictive interval plots were used to account for expected variation in future studies and to contextualize the certainty of evidence. All statistical tests were two-tailed, and significance was set at *p* < 0.05.

## Results

### Characteristics of included studies

**Fig. 1:**
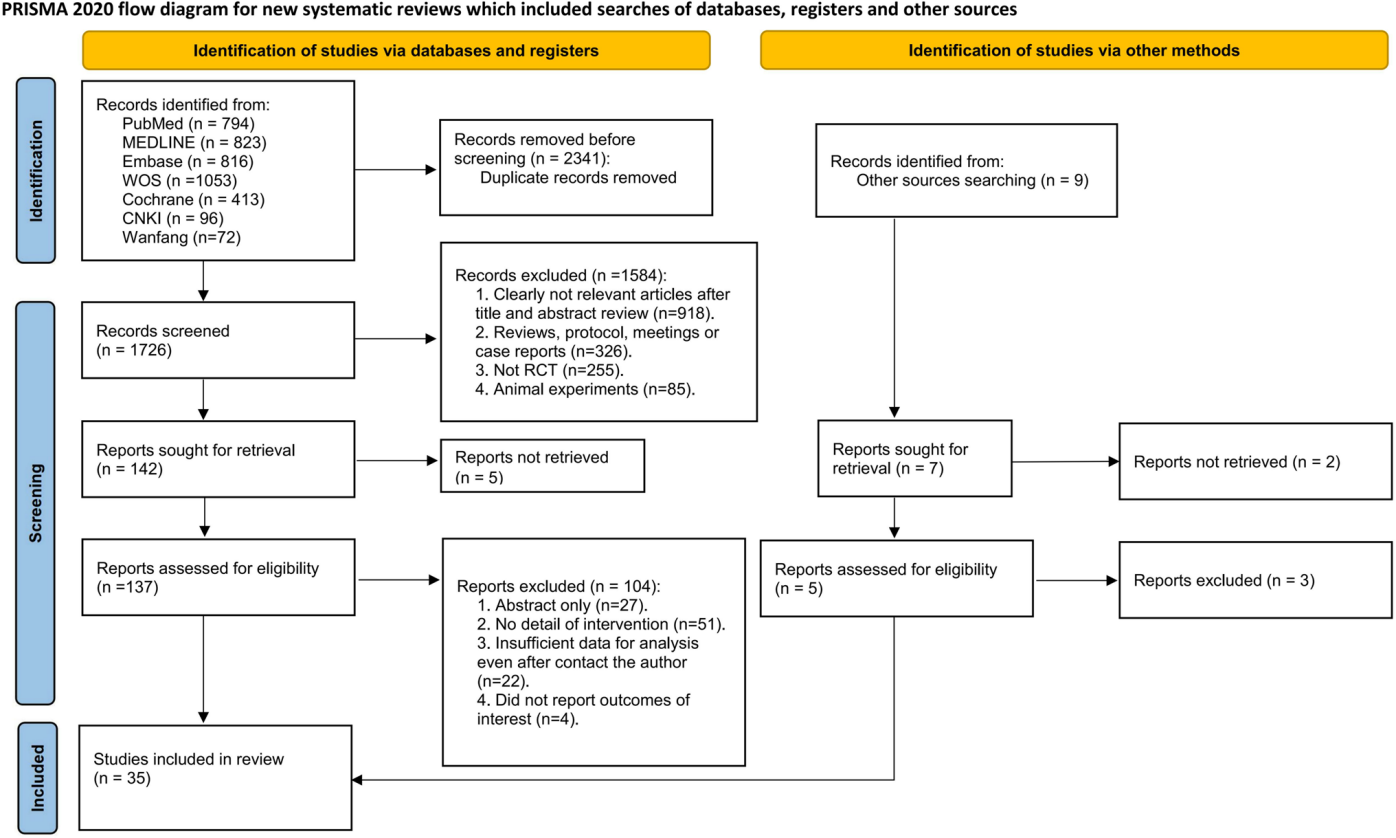
PRISMA flow diagram of the search process for studies

The initial electronic database search identified 4067 articles. After removing 2341 duplicates, 1726 articles were screened based on titles and abstracts, resulting in the exclusion of 1584 studies. Subsequently, 142 full-text articles underwent eligibility evaluation, with 35 RCTs involving 2919 cancer patients receiving chemotherapy ultimately included in this systematic review and network meta-analysis (Fig. 1) [20–54].

The included studies were published between 2007 and 2025, with a median publication year of 2021. The sample sizes of these studies ranged from 30 to 334 participants, with a median of 79 participants per study. The mean age of participants ranged from 33.0 to 57.5 years, with a median of 54.6 years. Regarding the TCM non-pharmacological interventions, seven studies utilized acupuncture, five used auricular therapy, eight applied manual acupoint therapy, twelve implemented mind–body exercise therapy, and five employed moxibustion. Comprehensive characteristics of the included studies are provided in Supplementary Table 6.1. In addition, the extracted mean ± SD values for the original outcome scales across study arms are presented in Supplementary Table 6.2. In addition, granular intervention characteristics for each trial arm, including WHO-standardized acupoint prescriptions or exercise protocols, session parameters, provider credentials, fidelity procedures, adherence monitoring, and co-interventions, are summarized in Supplementary File 3.

### Results of network meta-analysis

Network consistency was assessed for each outcome using the design-by-treatment interaction model, with global inconsistency results reported in Supplementary File 4. For outcomes showing evidence of global inconsistency, local inconsistency was further examined using node-splitting analyses, with results reported in Supplementary File 5.

### Quality of life (QoL)

**Fig. 2:**
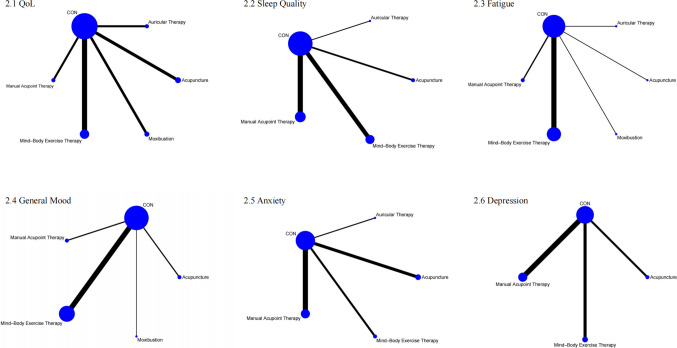
Network plot comparison of outcomes. (1) QoL, (2) sleep quality, (3) fatigue, (4) general mood, (5) anxiety, (6) depression

**Fig. 3:**
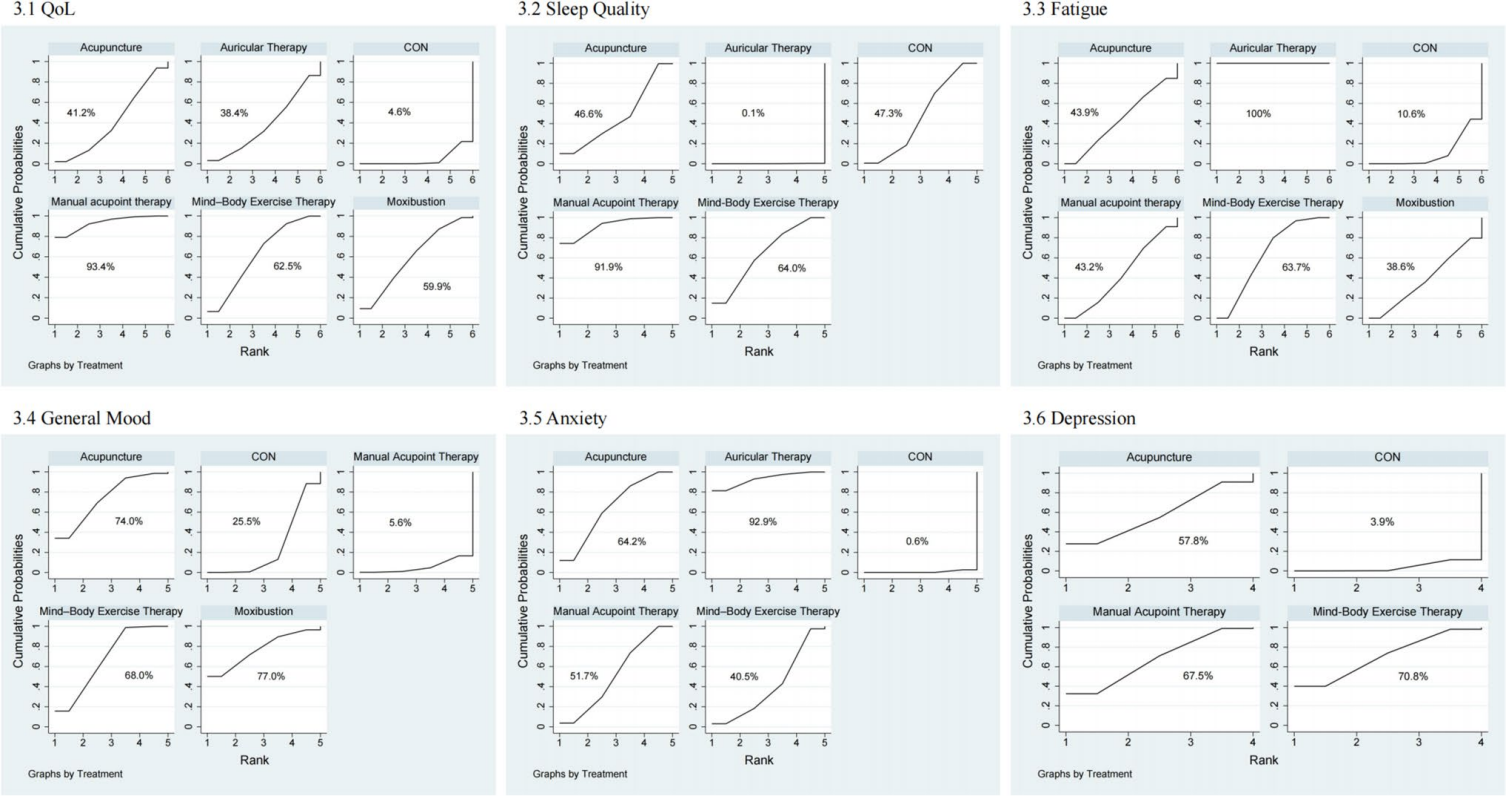
SUCRA probability ranking plot of outcomes. (1) QoL, (2) sleep quality, (3) fatigue, (4) general mood, (5) anxiety, (6) depression

Twenty-nine studies (*n* = 2061) assessed the effects of various TCM interventions on QoL. Not all included trials reported QoL, and therefore the QoL sample size is smaller than the total number of participants included in the review. Figure 2 illustrates the direct comparisons and sample sizes among interventions. According to SUCRA rankings (Fig. 3), the top three interventions enhancing QoL were manual acupoint therapy (63.4%), mind–body exercise therapy (62.5%), and moxibustion (59.9%). Compared to control (CON), manual acupoint therapy (SMD = 2.29, 95% CI = 0.95 to 3.63), mind–body exercise therapy (SMD = 1.29, 95% CI = 0.46 to 2.11), and moxibustion (SMD = 1.23, 95% CI = 0.07 to 2.40) significantly improved QoL (Table 1). We noted that the observed SMD of 2.29 for manual acupoint therapy in improving QoL corresponds to an estimated increase of 10 points on the EORTC QLQ-C30, exceeding the MCID of 10 points, indicating potential clinical benefit. Global inconsistency testing and, where applicable, node-splitting results for QoL are provided in Supplementary Files 4 and 5.

**Table 1:** League table of efficacy. QoL

Manual acupoint therapy					
1.00 (−0.57, 2.57)	Mind–body exercise therapy				
1.06 (−0.72, 2.83)	0.06 (−1.37, 1.48)	Moxibustion			
1.48 (−0.23, 3.19)	0.48 (−0.86, 1.83)	0.43 (−1.15, 2.01)	Acupuncture		
1.54 (−0.34, 3.41)	0.54 (−1.01, 2.08)	0.48 (−1.27, 2.23)	0.05 (−1.63, 1.74)	Auricular therapy	
2.29 (0.95, 3.63)	1.29 (0.46, 2.11)	1.23 (0.07, 2.40)	0.81 (−0.26, 1.87)	0.75 (−0.56, 2.06)	CON

### Sleep quality

Fourteen studies (*n* = 1084) evaluated sleep quality. Figure 2 illustrates the comparative network. SUCRA rankings (Fig. 3) indicated that manual acupoint therapy (91.9%), mind–body exercise therapy (64.0%), and acupuncture (46.6%) were most effective in improving sleep quality. Auricular therapy ranked lowest. Relative to auricular therapy, significant improvements in sleep quality were observed with manual acupoint therapy (SMD = −9.92, 95% CI = −14.83 to −5.01), mind–body exercise therapy (SMD = −8.71, 95% CI = −13.68 to −3.74), and acupuncture (SMD = −7.81, 95% CI = −13.33 to −2.29) (Table 2). The SMD of −9.92 for manual acupoint therapy corresponds to an estimated PSQI reduction of 7.6 points, exceeding the MCID of 3 points, suggesting clinical benefit. No statistically significant global inconsistency was detected for the sleep quality network (Supplementary File 4).

**Table 2:** League table of efficacy. Sleep quality

Manual acupoint therapy				
−1.21 (−3.85, 1.43)	Mind–body exercise therapy			
−1.79 (−3.57, −0.00)	−0.58 (−2.53, 1.37)	CON		
−2.11 (−5.67, 1.46)	−0.90 (−4.55, 2.76)	−0.32 (−3.41, 2.77)	Acupuncture	
−9.92 (−14.83, −5.01)	−8.71 (−13.68, −3.74)	−8.13 (−12.70, −3.56)	−7.81 (−13.33, −2.29)	Auricular therapy

### Fatigue

Thirteen studies (*n* = 1120) assessed fatigue outcomes. Network comparisons are displayed in Fig. 2. Auricular therapy demonstrated the highest SUCRA score (100%), followed by mind–body exercise therapy (63.7%) and acupuncture (43.9%) (Fig. 3). Auricular therapy significantly reduced fatigue compared to CON (SMD = −8.13, 95% CI = −10.15 to −6.11), moxibustion (SMD = −7.62, 95% CI = −10.10 to −5.14), manual acupoint therapy (SMD = −7.48, 95% CI = −9.76 to −5.20), acupuncture (SMD = −7.47, 95% CI = −9.93 to −5.00), and mind–body exercise therapy (SMD = −7.09, 95% CI = −9.17 to −5.00) (Table 3). The SMD of −8.13 for auricular therapy corresponds to an estimated reduction of 5.6 points on the BFI, exceeding the MCID of 1.3 points, suggesting clinical benefit. Global inconsistency testing did not indicate significant inconsistency for fatigue (Supplementary File 4).

**Table 3:** League table of efficacy. Fatigue

Auricular therapy					
−7.09 (−9.17, −5.00)	Mind–body exercise therapy				
−7.47 (−9.93, −5.00)	−0.38 (−1.88, 1.12)	Acupuncture			
−7.48 (−9.76, −5.20)	−0.39 (−1.56, 0.77)	−0.01 (−1.77, 1.74)	Manual acupoint therapy		
−7.62 (−10.10, −5.14)	−0.53 (−2.06, 0.99)	−0.16 (−2.17, 1.86)	−0.14 (−1.92, 1.64)	Moxibustion	
−8.13 (−10.15, −6.11)	−1.04 (−1.55, −0.53)	−0.66 (−2.07, 0.74)	−0.65 (−1.69, 0.40)	−0.51 (−1.94, 0.93)	CON

### General mood

Fourteen studies (*n* = 1388) reported on general mood outcomes. General mood reflected overall emotional functioning derived from validated quality-of-life instruments rather than symptom-specific anxiety or depression scales. Comparative networks are shown in Fig. 2. According to SUCRA rankings (Fig. 3), moxibustion (77.0%), acupuncture (74.0%), and mind–body exercise therapy (68.0%) were most effective in improving general mood. Mind–body exercise therapy significantly enhanced mood relative to manual acupoint therapy (SMD = 1.34, 95% CI = 0.14 to 2.55) and CON (SMD = 0.72, 95% CI = 0.20 to 1.24) (Table 4). The SMD of 1.34 for mind–body exercise therapy corresponds to an estimated increase of 3.8 points on the EORTC QLQ-C30 Emotional Functioning domain, exceeding the MCID of 3 points, indicating clinical benefit. Global inconsistency testing did not indicate significant inconsistency for general mood (Supplementary File 4).

**Table 4:** League table of efficacy. General mood

Moxibustion				
0.16 (−1.78, 2.10)	Acupuncture			
0.35 (−1.33, 2.02)	0.18 (−1.05, 1.41)	Mind–body exercise therapy		
1.06 (−0.53, 2.65)	0.90 (−0.21, 2.01)	0.72 (0.20, 1.24)	CON	
1.69 (−0.24, 3.62)	1.52 (−0.03, 3.08)	1.34 (0.14, 2.55)	0.63 (−0.46, 1.71)	Manual acupoint therapy

### Anxiety

Eleven studies (*n* = 759) evaluated anxiety outcomes. Figure 2 demonstrates the network. Auricular therapy ranked highest in SUCRA (92.9%), followed by acupuncture (64.2%) and manual acupoint therapy (51.7%) (Fig. 3). Compared to CON, auricular therapy (SMD = −1.53, 95% CI = −2.48 to −0.58), acupuncture (SMD = −0.94, 95% CI = −1.50 to −0.38), and manual acupoint therapy (SMD = −0.79, 95% CI = −1.22 to −0.36) significantly improved anxiety outcomes (Table 5). Global inconsistency testing did not indicate significant inconsistency for anxiety (Supplementary File 4).

**Table 5:** League table of efficacy. Anxiety

Auricular therapy				
−0.59 (−1.69, 0.52)	Acupuncture			
−0.74 (−1.78, 0.30)	−0.15 (−0.85, 0.55)	Manual acupoint therapy		
−0.89 (−2.04, 0.25)	−0.31 (−1.15, 0.54)	−0.16 (−0.93, 0.61)	Mind–body exercise therapy	
−1.53 (−2.48, −0.58)	−0.94 (−1.50, −0.38)	−0.79 (−1.22, −0.36)	−0.63 (−1.28, 0.01)	CON

### Depression

Ten studies (*n* = 710) analyzed depression outcomes. Network connections are depicted in Fig. 2. SUCRA rankings (Fig. 3) identified mind–body exercise therapy (70.8%), manual acupoint therapy (67.5%), and acupuncture (57.8%) as most effective. Compared to CON, significant reductions in depression were observed with mind–body exercise therapy (SMD = −0.92, 95% CI = −1.79 to −0.05) and manual acupoint therapy (SMD = −0.87, 95% CI = −1.56 to −0.18) (Table 6). Global inconsistency testing and, where applicable, node-splitting results for depression are provided in Supplementary Files 4 and 5.

**Table 6:** League table of efficacy. Depression

Mind–body exercise therapy			
−0.05 (−1.16, 1.06)	Manual acupoint therapy		
−0.16 (−1.56, 1.23)	−0.11 (−1.40, 1.18)	Acupuncture	
−0.92 (−1.79, −0.05)	−0.87 (−1.56, −0.18)	−0.76 (−1.85, 0.33)	CON

### Risk of bias and publication bias

Risk of bias across the 35 included randomized controlled trials was assessed using the revised RoB 2. Overall, 15 studies were rated as low risk, 15 as having some concerns, and 5 as high risk. For the randomization process, 25 studies were judged as low risk, 8 as having some concerns, and 2 as high risk. In the domain of deviations from intended interventions, 28 studies were assessed as low risk, 4 as having some concerns, and 3 as high risk. Regarding missing outcome data, 23 studies were rated as low risk, 9 as having some concerns, and 3 as high risk. Outcome measurement was judged as low risk in 32 studies, with 2 rated as having some concerns and 1 as high risk. All studies were considered low risk for selective reporting. Full details are available in Supplementary File 2.

Publication bias was explored using comparison-adjusted funnel plots (Supplementary File 7). The distribution of studies around the vertical axis showed varying asymmetry, indicating potential small-study effects. Notably, funnel plots for all six outcome domains (Figures S4.1–S4.6) displayed some asymmetry. Egger’s test identified significant bias for the quality of life outcome (*p* < 0.05), suggesting cautious interpretation. For other outcomes, Egger’s tests were non-significant (*p* > 0.05), indicating no clear evidence of publication bias.

## Discussion

This network meta-analysis integrated data from 35 randomized controlled trials involving a total of 2919 cancer patients undergoing chemotherapy, aiming to compare the relative efficacy of five non-pharmacological interventions derived from TCM in improving quality of life and emotional well-being. Several important findings emerged. Importantly, treatment rankings based on SUCRA values should be interpreted as relative comparisons among TCM modalities rather than as indicators of absolute superiority. First, manual acupoint therapy demonstrated the most robust effects in enhancing both quality of life and sleep quality. Among all included interventions, it ranked highest for improving overall HRQoL and exhibited superior benefits in sleep enhancement when compared with auricular therapy, acupuncture, and mind–body exercise therapy. Second, mind–body exercise therapy consistently ranked among the top three interventions across all outcomes except anxiety, with its most notable benefits observed in the domains of general mood and depression, where it achieved the highest effectiveness. It also demonstrated favorable performance in improving quality of life, sleep quality, and fatigue. Third, auricular therapy exhibited distinct advantages in alleviating chemotherapy-related fatigue and anxiety, emerging as the top-ranking intervention for fatigue reduction and also leading in anxiety relief. Collectively, these findings highlight the differentiated profiles and symptom-specific advantages of various TCM non-pharmacological therapies. Nevertheless, several comparisons yielded unusually large standardized mean differences, suggesting that pooled estimates may be influenced by small-study effects, indirect comparisons, and scale-related amplification rather than reflecting precise clinical magnitude. Accordingly, greater emphasis should be placed on relative rankings and directional consistency rather than absolute effect sizes. They provide a comprehensive evidence base for tailoring supportive care interventions to the individual needs of patients with cancer, promoting a more integrative, precise, and patient-centered approach to oncologic rehabilitation.

The growing emphasis on supportive care in oncology has brought increasing attention to non-pharmacological interventions rooted in TCM, particularly for addressing the complex symptom burden experienced by patients undergoing chemotherapy. Among these burdens, compromised quality of life remains a central concern, given its multidimensional impact on physical, emotional, and functional well-being [55]. In this context, our network meta-analysis identified manual acupoint therapy as the most effective intervention for enhancing quality of life among cancer patients receiving chemotherapy. This finding aligns with previous studies that have demonstrated the beneficial effects of Tuina massage and body acupressure on fatigue, pain, and emotional distress in oncology populations, although few have systematically ranked its comparative efficacy against other TCM modalities. The current analysis not only confirms these benefits but also extends prior evidence by demonstrating the superior position of manual acupoint therapy in a comprehensive treatment network [56]. From a clinical perspective, the observed improvements in quality of life exceeded the established minimal clinically important difference of 10 points for the EORTC QLQ-C30, suggesting that the benefits are likely to be clinically meaningful. Notably, manual acupoint therapy also emerged as the top-ranking intervention for improving sleep quality, a common and debilitating complaint among cancer patients. Sleep disturbances in this population are often multifactorial, arising from chemotherapy-induced nausea, pain, anxiety, and circadian disruption [57, 58]. The dual efficacy of manual acupoint therapy in enhancing both quality of life and sleep may be attributed to its multimodal mechanisms of action. Mechanistically, manual stimulation of specific acupoints has been shown to modulate autonomic nervous system activity, particularly by enhancing parasympathetic tone and reducing sympathetic overactivity, thereby promoting relaxation and sleep initiation [59]. Additionally, acupressure and massage may trigger endogenous opioid release, reduce pro-inflammatory cytokines, and improve microcirculation, contributing to pain relief and emotional stabilization. These physiological effects likely translate into broader improvements in well-being and functional capacity, thereby explaining the observed gains in HRQoL [60]. However, intervention protocols, outcome instruments, and treatment intensity varied considerably across trials, and many sleep-related analyses were informed by relatively small sample sizes, which may partially account for the magnitude of standardized effects observed.

Among the spectrum of TCM-based non-pharmacological approaches, mind–body exercise therapy represents a particularly integral modality, owing to its holistic emphasis on the integration of physical movement, breath regulation, and mental focus. These practices, exemplified by Tai Chi and Qigong, are increasingly incorporated into integrative oncology care for their potential to address both physiological and psychological sequelae of cancer treatment [61]. In the present study, mind–body exercise therapy emerged as one of the most consistently effective interventions, ranking within the top three across nearly all outcome domains. Notably, it achieved the highest effectiveness in improving general mood and reducing depressive symptoms, and demonstrated strong benefits for enhancing quality of life, sleep quality, and mitigating fatigue. The magnitude of improvement in depressive symptoms exceeded the commonly accepted minimal clinically important difference of 3 points on the HADS, supporting the potential clinical relevance of these findings. The prominence of this modality in improving general mood supports its role in enhancing broader emotional functioning rather than targeting isolated affective symptoms. These findings are broadly consistent with prior meta-analyses that have shown positive effects of Tai Chi and Qigong on psychological outcomes and functional status in cancer populations [62, 63]. However, compared with conventional pairwise meta-analyses, the effect sizes observed in this network meta-analysis were generally larger, which may reflect differences in analytic framework, comparator structure, and the inclusion of smaller trials. By employing a network meta-analytic framework, our study substantiates and expands upon earlier evidence, while also highlighting the need for cautious interpretation.

By employing a network meta-analytic framework, our study substantiates and expands upon earlier evidence, highlighting the comparative advantage of mind–body exercise therapy in promoting multidimensional recovery in chemotherapy patients. The therapeutic benefits of this modality may be explained by its unique capacity to modulate the hypothalamic–pituitary–adrenal (HPA) axis and autonomic nervous system, both of which are dysregulated under chronic stress and chemotherapy exposure. Mind–body exercise has been shown to reduce cortisol levels, enhance vagal tone, and improve sleep efficiency through entrainment of circadian rhythms [64]. Additionally, the meditative and low-impact aerobics components improve cardiorespiratory fitness and mood by promoting neuroplasticity, increasing brain-derived neurotrophic factor (BDNF), and reducing systemic inflammation [65]. These mechanisms may collectively underpin its broad therapeutic profile observed in this study.

However, it is noteworthy that mind–body exercise therapy ranked relatively lower in the domain of anxiety reduction, despite its favorable performance in other outcomes. This divergence may stem from the nature and intensity of anxiety experienced during chemotherapy, which often requires targeted cognitive or neurobehavioral interventions to address anticipatory distress, existential fears, and procedural anxiety. Unlike acupoint-based therapies, mind–body exercise may exert more gradual and nonspecific effects, which could be less immediate or insufficiently potent for mitigating acute anxiety symptoms [66]. Furthermore, variability in intervention fidelity and patient adherence may also influence its efficacy in this specific domain. Future studies should explore whether combining mind–body exercise with acupoint-based or psychological therapies could yield synergistic benefits for anxiety relief in cancer patients.

A notable and distinctive finding of this study was the superior performance of auricular therapy in alleviating fatigue and anxiety symptoms among cancer patients undergoing chemotherapy. Specifically, this modality ranked highest in reducing fatigue severity and demonstrated the most pronounced anxiolytic effect compared to other TCM-based non-pharmacological interventions. These findings are partially aligned with previous literature that has reported beneficial outcomes of auricular acupuncture and acupressure in the management of chemotherapy-related symptoms, including insomnia, fatigue, and mood disturbances. However, unlike prior studies which have generally examined auricular techniques as adjuncts or within multimodal protocols, our network meta-analysis isolates and compares their standalone efficacy, thereby providing a more definitive understanding of their relative therapeutic value [67]. The estimated reductions in fatigue exceeded the minimal clinically important difference of 1.3 points on the Brief Fatigue Inventory, suggesting potential clinical benefit, although the exceptionally large pooled effects may reflect small-study effects. The prominent efficacy of auricular therapy in addressing fatigue and anxiety may be mechanistically explained by its neurophysiological effects on the auricular branch of the vagus nerve (ABVN). Stimulation of auricular acupoints is known to activate afferent fibers that project to the nucleus tractus solitarius (NTS), thereby modulating parasympathetic output and promoting systemic homeostasis. This vagal afferent stimulation has been associated with reduced sympathetic arousal, dampened hypothalamic–pituitary–adrenal (HPA) axis activity, and enhanced regulation of neurotransmitters such as serotonin and gamma-aminobutyric acid (GABA), all of which are implicated in the pathophysiology of anxiety and cancer-related fatigue [68]. Moreover, auricular therapy is minimally invasive, easy to administer, and has the advantage of continuous stimulation through press seeds or embedded needles, potentially prolonging its therapeutic effect beyond active sessions. Nevertheless, the exceptionally large pooled estimates for fatigue outcomes may reflect small-study effects and should be interpreted conservatively.

Interestingly, despite its effectiveness in mitigating anxiety and fatigue, auricular therapy ranked lower for improving overall quality of life and sleep quality. This apparent discrepancy may be attributed to the modality’s relatively narrow therapeutic scope, as it primarily targets neuroautonomic regulation rather than engaging multiple biopsychosocial dimensions. In contrast to more holistic interventions like mind–body exercise therapy or manual acupoint therapy—which simultaneously modulate physical, emotional, and behavioral domains—auricular therapy may not sufficiently influence the broader contributors to sleep disturbance or psychosocial functioning. Furthermore, the passive nature of this intervention may limit its ability to induce empowerment or foster meaningful patient engagement, factors that are increasingly recognized as essential to enhancing quality of life in cancer care. This divergence underscores the need for personalized and multimodal treatment strategies that align with the complex and multidimensional needs of chemotherapy patients.

These findings carry important practical implications for integrative oncology. As supportive care increasingly focuses on improving quality of life and psychological well-being, identifying effective and low-risk non-pharmacological strategies is essential. This network meta-analysis provides robust comparative evidence, enabling clinicians to align specific TCM-based interventions with patients’ dominant symptoms and personal preferences. Manual acupoint therapy may be especially suitable for patients with significant sleep disturbances or impaired quality of life, while mind–body exercise therapy offers broad benefits across multiple domains and may serve as a cornerstone of comprehensive survivorship care. Auricular therapy, with its simplicity and notable effects on fatigue and anxiety, may be particularly useful in resource-limited settings or for patients with limited physical capacity. Importantly, these interventions are generally well-tolerated and culturally acceptable, offering viable alternatives or adjuncts to conventional treatments. Our findings support their inclusion in multidisciplinary care and provide a foundation for future research and guideline development in culturally diverse healthcare settings.

This study possesses several notable strengths. First, it is the first network meta-analysis to comprehensively compare the efficacy of five distinct TCM non-pharmacological therapies in improving quality of life, sleep, fatigue, and emotional outcomes among cancer patients undergoing chemotherapy. By integrating both direct and indirect evidence across 35 randomized controlled trials, this approach offers a hierarchical comparison that overcomes some limitations of traditional pairwise meta-analyses. At the same time, important limitations warrant emphasis. Substantial heterogeneity in intervention protocols and background supportive care likely contributed to variability in effect estimates. Psychological outcomes were informed by modest sample sizes, limiting precision. Although Chinese databases were searched, many eligible Chinese-language trials lacked sufficient quantitative data for inclusion, which may have introduced selection bias. Finally, the presence of implausibly large standardized mean differences in several networks suggests potential overestimation driven by small-study effects and indirect evidence. These limitations highlight the need for larger, rigorously designed, head-to-head trials with standardized protocols and outcome reporting to validate and refine these findings.

## Conclusion

This network meta-analysis of 35 RCTs demonstrates that TCM-based non-pharmacological therapies offer targeted benefits for cancer patients undergoing chemotherapy. Manual acupoint therapy showed the strongest effects on quality of life and sleep. Mind–body exercise therapy provided broad improvements, especially for mood and depression, while auricular therapy was most effective for fatigue and anxiety. These findings support the use of symptom-specific TCM interventions as safe, culturally appropriate, and patient-centered options to enhance supportive cancer care. Integration into routine practice may optimize outcomes and improve survivorship. Further high-quality trials are needed to confirm these results and guide personalized treatment strategies.

## Supplementary information

Below is the link to the electronic supplementary material.

ESM 1 (DOCX 1.45 MB)

## Data Availability

All data generated or analysed during this study are included in this published article and its supplementary information files.
