# Supplementary material for: Comparative effectiveness of traditional Chinese non-pharmacological therapies for chemotherapy-related symptoms in cancer patients: a systematic review and network meta-analysis
Source: Support Care Cancer. 2026 Jul 27;34(8):804. doi: 10.1007/s00520-026-10990-7 (PMC13407933; doi:10.1007/s00520-026-10990-7)
Supplement: Supplementary file 1 — (DOCX 1.45 MB) [file 520_2026_10990_MOESM1_ESM.docx]

**Supplementary**

Supplementary 1: Search Strategy

***1.1 Search Strategy (Pubmed):***

| #18 | Search: (((((((((((((((((Neoplasms[MeSH Terms]) OR (Tumors[Title/Abstract])) OR (Neoplasia[Title/Abstract])) OR (Neoplasias[Title/Abstract])) OR (Neoplasm[Title/Abstract])) OR (Tumor[Title/Abstract])) OR (Cancer[Title/Abstract])) OR (Cancers[Title/Abstract])) OR ("Malignant Neoplasm"[Title/Abstract])) OR (Malignancy[Title/Abstract])) OR (Malignancies[Title/Abstract])) OR ("Malignant Neoplasms"[Title/Abstract])) OR ("Benign Neoplasms"[Title/Abstract])) OR ("Benign Neoplasm"[Title/Abstract])) OR ("Cancer Survivor"[Title/Abstract])) AND ((((((((( ("Acupuncture"[Mesh]) OR ((((((((((((((((((((((((((((((Pharmacopuncture[Title/Abstract]) OR (Massage[Title/Abstract])) OR (Zone Therapy[Title/Abstract])) OR (Therapies, Zone[Title/Abstract])) OR (Zone Therapies[Title/Abstract])) OR (Therapy, Zone[Title/Abstract])) OR (Massage Therapy[Title/Abstract])) OR (Massage Therapies[Title/Abstract])) OR (Therapies, Massage[Title/Abstract])) OR (Therapy, Massage[Title/Abstract])) OR (Tuina[Title/Abstract])) OR (Qigong[Title/Abstract])) OR (Ch'i Kung[Title/Abstract])) OR (Qi Gong[Title/Abstract])) OR (Baduanjin[Title/Abstract])) OR (Wuqinxi[Title/Abstract])) OR (Liuzijue[Title/Abstract])) OR (Yi Jin Jing[Title/Abstract])) OR (Tai Ji[Title/Abstract])) OR (Tai-ji[Title/Abstract])) OR (Tai Chi[Title/Abstract])) OR (Chi, Tai[Title/Abstract])) OR (Tai Chi Chuan[Title/Abstract])) OR (Taiji[Title/Abstract])) OR (Taijiquan[Title/Abstract])) OR (T'ai Chi[Title/Abstract])) OR (Tai Ji Quan[Title/Abstract])) OR (Ji Quan, Tai[Title/Abstract])) OR (Quan, Tai Ji[Title/Abstract])) OR (Traditional Chinese medicine emotional therapy[Title/Abstract]))) OR (Moxibustion[MeSH Terms])) OR (Mind-Body Therapies[MeSH Terms])) OR (Tai Ji[MeSH Terms])) OR (Qigong[MeSH Terms])) OR (Medicine, Chinese Traditional[MeSH Terms])) OR (moxibustion[Title/Abstract])) OR ("Chinese massage"[Title/Abstract])) OR ("traditional Chinese medicine"[Title/Abstract]))) AND (((((((((((((((((((Depression[MeSH Terms]) OR (Anxiety[MeSH Terms])) OR (Mood Disorders[MeSH Terms])) OR (Sleep[MeSH Terms])) OR (Quality of Life[MeSH Terms])) OR (depression[Title/Abstract])) OR (depressive[Title/Abstract])) OR (depress*[Title/Abstract])) OR (anxiety[Title/Abstract])) OR (anxious[Title/Abstract])) OR (anxi*[Title/Abstract])) OR (mood[Title/Abstract])) OR (emotion*[Title/Abstract])) OR ("sleep quality"[Title/Abstract])) OR (insomnia[Title/Abstract])) OR ("quality of life"[Title/Abstract])) OR (QoL[Title/Abstract])) OR (wellbeing[Title/Abstract])) OR (well-being[Title/Abstract]))) AND ((randomized controlled trial[pt] OR controlled clinical trial[pt] OR randomized[tiab] OR placebo[tiab] OR drug therapy[sh] OR randomly[tiab] OR trial[tiab] OR groups[tiab]) NOT (animals[mh] NOT humans[mh])) |
| --- | --- |
| #17 | Search: (randomized controlled trial[pt] OR controlled clinical trial[pt] OR randomized[tiab] OR placebo[tiab] OR drug therapy[sh] OR randomly[tiab] OR trial[tiab] OR groups[tiab]) NOT (animals[mh] NOT humans[mh]) |
| #16 | Search: ((((((((((((((((((Depression[MeSH Terms]) OR (Anxiety[MeSH Terms])) OR (Mood Disorders[MeSH Terms])) OR (Sleep[MeSH Terms])) OR (Quality of Life[MeSH Terms])) OR (depression[Title/Abstract])) OR (depressive[Title/Abstract])) OR (depress*[Title/Abstract])) OR (anxiety[Title/Abstract])) OR (anxious[Title/Abstract])) OR (anxi*[Title/Abstract])) OR (mood[Title/Abstract])) OR (emotion*[Title/Abstract])) OR ("sleep quality"[Title/Abstract])) OR (insomnia[Title/Abstract])) OR ("quality of life"[Title/Abstract])) OR (QoL[Title/Abstract])) OR (wellbeing[Title/Abstract])) OR (well-being[Title/Abstract]) |
| #15 | Search: (depression[Title/Abstract])) OR (depressive[Title/Abstract])) OR (depress*[Title/Abstract])) OR (anxiety[Title/Abstract])) OR (anxious[Title/Abstract])) OR (anxi*[Title/Abstract])) OR (mood[Title/Abstract])) OR (emotion*[Title/Abstract])) OR ("sleep quality"[Title/Abstract])) OR (insomnia[Title/Abstract])) OR ("quality of life"[Title/Abstract])) OR (QoL[Title/Abstract])) OR (wellbeing[Title/Abstract])) OR (well-being[Title/Abstract]) |
| #14 | Search: Quality of Life[MeSH Terms] |
| #13 | Search: Mood Disorders[MeSH Terms] |
| #12 | Search: Anxiety[MeSH Terms] |
| #11 | Search: Depression[MeSH Terms] |
| #10 | Search:(((((((( ("Acupuncture"[Mesh]) OR ((((((((((((((((((((((((((((((Pharmacopuncture[Title/Abstract]) OR (Massage[Title/Abstract])) OR (Zone Therapy[Title/Abstract])) OR (Therapies, Zone[Title/Abstract])) OR (Zone Therapies[Title/Abstract])) OR (Therapy, Zone[Title/Abstract])) OR (Massage Therapy[Title/Abstract])) OR (Massage Therapies[Title/Abstract])) OR (Therapies, Massage[Title/Abstract])) OR (Therapy, Massage[Title/Abstract])) OR (Tuina[Title/Abstract])) OR (Qigong[Title/Abstract])) OR (Ch'i Kung[Title/Abstract])) OR (Qi Gong[Title/Abstract])) OR (Baduanjin[Title/Abstract])) OR (Wuqinxi[Title/Abstract])) OR (Liuzijue[Title/Abstract])) OR (Yi Jin Jing[Title/Abstract])) OR (Tai Ji[Title/Abstract])) OR (Tai-ji[Title/Abstract])) OR (Tai Chi[Title/Abstract])) OR (Chi, Tai[Title/Abstract])) OR (Tai Chi Chuan[Title/Abstract])) OR (Taiji[Title/Abstract])) OR (Taijiquan[Title/Abstract])) OR (T'ai Chi[Title/Abstract])) OR (Tai Ji Quan[Title/Abstract])) OR (Ji Quan, Tai[Title/Abstract])) OR (Quan, Tai Ji[Title/Abstract])) OR (Traditional Chinese medicine emotional therapy[Title/Abstract]))) OR (Moxibustion[MeSH Terms])) OR (Mind-Body Therapies[MeSH Terms])) OR (Tai Ji[MeSH Terms])) OR (Qigong[MeSH Terms])) OR (Medicine, Chinese Traditional[MeSH Terms])) OR (moxibustion[Title/Abstract])) OR (Acupressure[Title/Abstract])) OR ("Gua sha"[Title/Abstract])) OR ("Chinese massage"[Title/Abstract])) OR ("traditional Chinese medicine"[Title/Abstract]) |
| #9 | Search: (Pharmacopuncture[Title/Abstract]) OR (Massage[Title/Abstract])) OR (Zone Therapy[Title/Abstract])) OR (Therapies, Zone[Title/Abstract])) OR (Zone Therapies[Title/Abstract])) OR (Therapy, Zone[Title/Abstract])) OR (Massage Therapy[Title/Abstract])) OR (Massage Therapies[Title/Abstract])) OR (Therapies, Massage[Title/Abstract])) OR (Therapy, Massage[Title/Abstract])) OR (Tuina[Title/Abstract])) OR (Qigong[Title/Abstract])) OR (Ch'i Kung[Title/Abstract])) OR (Qi Gong[Title/Abstract])) OR (Baduanjin[Title/Abstract])) OR (Wuqinxi[Title/Abstract])) OR (Liuzijue[Title/Abstract])) OR (Yi Jin Jing[Title/Abstract])) OR (Tai Ji[Title/Abstract])) OR (Tai-ji[Title/Abstract])) OR (Tai Chi[Title/Abstract])) OR (Chi, Tai[Title/Abstract])) OR (Tai Chi Chuan[Title/Abstract])) OR (Taiji[Title/Abstract])) OR (Taijiquan[Title/Abstract])) OR (T'ai Chi[Title/Abstract])) OR (Tai Ji Quan[Title/Abstract])) OR (Ji Quan, Tai[Title/Abstract])) OR (Quan, Tai Ji[Title/Abstract])) OR (Traditional Chinese medicine emotional therapy[Title/Abstract]))) OR (Tai Ji[MeSH Terms])) OR (Qigong[MeSH Terms])) OR (Medicine, Chinese Traditional[MeSH Terms])) OR (moxibustion[Title/Abstract])) OR (Acupressure[Title/Abstract])) OR ("Gua sha"[Title/Abstract])) OR ("Chinese massage"[Title/Abstract])) OR ("traditional Chinese medicine"[Title/Abstract]) |
| #8 | Search: Medicine, Chinese Traditional[MeSH Terms] |
| #7 | Search: Qigong[MeSH Terms] |
| #6 | Search: Tai Ji[MeSH Terms] |
| #5 | Search: Mind-Body Therapies[MeSH Terms] |
| #4 | Search: "Acupuncture"[Mesh] |
| #3 | Search: ((((((((((((((Neoplasms[MeSH Terms]) OR (Tumors[Title/Abstract])) OR (Neoplasia[Title/Abstract])) OR (Neoplasias[Title/Abstract])) OR (Neoplasm[Title/Abstract])) OR (Tumor[Title/Abstract])) OR (Cancer[Title/Abstract])) OR (Cancers[Title/Abstract])) OR ("Malignant Neoplasm"[Title/Abstract])) OR (Malignancy[Title/Abstract])) OR (Malignancies[Title/Abstract])) OR ("Malignant Neoplasms"[Title/Abstract])) OR ("Benign Neoplasms"[Title/Abstract])) OR ("Benign Neoplasm"[Title/Abstract])) OR ("Cancer Survivor"[Title/Abstract]) |
| #2 | Search:  (Tumors[Title/Abstract])) OR (Neoplasia[Title/Abstract])) OR (Neoplasias[Title/Abstract])) OR (Neoplasm[Title/Abstract])) OR (Tumor[Title/Abstract])) OR (Cancer[Title/Abstract])) OR (Cancers[Title/Abstract])) OR ("Malignant Neoplasm"[Title/Abstract])) OR (Malignancy[Title/Abstract])) OR (Malignancies[Title/Abstract])) OR ("Malignant Neoplasms"[Title/Abstract])) OR ("Benign Neoplasms"[Title/Abstract])) OR ("Benign Neoplasm"[Title/Abstract])) OR ("Cancer Survivor"[Title/Abstract]) |
| #1 | Search: Neoplasms[MeSH Terms] |

***1.2 Search Strategy (Embase):***

1 (tumor$ or tumour$ or neoplas$ or cancer$ or malignan$ or "cancer survivor$").mp.

2 exp neoplasm/

3 (acupuncture or pharmacopuncture or moxibustion or acupressure or tuina or massage or "massage therapy" or "chinese massage" or "gua sha" or qigong or "qi gong" or "chi kung" or "tai chi" or tai ji or taiji or taijiquan or baduanjin or wuqinxi or liuzijue or "yi jin jing" or "mind body" or "mind-body" or "traditional chinese medicine").mp.

4 exp acupuncture/

5 exp moxibustion/

6 exp acupressure/

7 exp massage/

8 exp mind body therapy/

9 exp tai chi/

10 exp qigong/

11 exp traditional chinese medicine/

12 (depress$ or depressive$ or anxiety or anxious or anxi$ or mood or emotion$ or sleep or insomnia or "sleep quality" or "quality of life" or qol or wellbeing or well-being).mp.

13 exp depression/

14 exp anxiety/

15 exp mood disorder/

16 exp sleep disorder/

17 exp quality of life/

18 randomized.ab.

19 randomly.ab.

20 trial.ti.

21 placebo.ab.

22 exp randomized controlled trial/

23 exp controlled clinical trial/

24 exp clinical trial/

25 (clinic$ adj2 trial).mp.

26 (random$ adj5 control$ adj5 trial$).mp.

27 randomi$.mp.

28 1 or 2

29 3 or 4 or 5 or 6 or 7 or 8 or 9 or 10 or 11

30 12 or 13 or 14 or 15 or 16 or 17

31 18 or 19 or 20 or 21 or 22 or 23 or 24 or 25 or 26 or 27

32 28 and 29 and 30 and 31

33 limit 32 to human

34 limit 33 to english language

***1.3 Search Strategy (Cochrane):***

#1 MeSH descriptor: [Neoplasms] explode all trees

#2 (tumor* or tumour* or neoplasm* or cancer* or malignan* or "cancer survivor*") in Trials (Word variations have been searched)

#3 MeSH descriptor: [Acupuncture Therapy] explode all trees

#4 MeSH descriptor: [Moxibustion] explode all trees

#5 MeSH descriptor: [Acupressure] explode all trees

#6 MeSH descriptor: [Massage] explode all trees

#7 MeSH descriptor: [Mind-Body Therapies] explode all trees

#8 MeSH descriptor: [Tai Ji] explode all trees

#9 MeSH descriptor: [Qigong] explode all trees

#10 MeSH descriptor: [Medicine, Chinese Traditional] explode all trees

#11 (acupuncture or pharmacopuncture or moxibustion or acupressure or tuina or massage or "chinese massage" or "gua sha" or qigong or "qi gong" or "chi kung" or "tai chi" or tai ji or taiji or taijiquan or baduanjin or wuqinxi or liuzijue or "yi jin jing" or "traditional chinese medicine") in Trials (Word variations have been searched)

#12 #3 or #4 or #5 or #6 or #7 or #8 or #9 or #10 or #11

#13 MeSH descriptor: [Depression] explode all trees

#14 MeSH descriptor: [Anxiety] explode all trees

#15 MeSH descriptor: [Mood Disorders] explode all trees

#16 MeSH descriptor: [Sleep Wake Disorders] explode all trees

#17 MeSH descriptor: [Quality of Life] explode all trees

#18 (depress* or anxiety or anxious or anxi* or mood or emotion* or sleep or insomnia or "sleep quality" or "quality of life" or QoL or wellbeing or well-being) in Trials (Word variations have been searched)

#19 #13 or #14 or #15 or #16 or #17 or #18

#20 #1 or #2

#21 #20 and #12 and #19

***1.4 Search Strategy (Web of Science):***

#13 #12 AND #11 AND #1

Indexes=SCI-EXPANDED, SSCI, A&HCI, CPCI-S, CPCI-SSH, BKCI-S, BKCI-SSH, ESCI, CCR-EXPANDED, IC

Timespan=All years

#12 #10 OR #9 OR #8 OR #7 OR #6 OR #5 OR #4 OR #3 OR #2

Indexes=SCI-EXPANDED, SSCI, A&HCI, CPCI-S, CPCI-SSH, BKCI-S, BKCI-SSH, ESCI, CCR-EXPANDED, IC

Timespan=All years

#11 TOPIC: ((“randomized controlled trial*” OR “controlled clinical trial” OR random* OR randomly OR trial OR “clinical trial*” OR “cross-over stud*” OR clinic*))

Indexes=SCI-EXPANDED, SSCI, A&HCI, CPCI-S, CPCI-SSH, BKCI-S, BKCI-SSH, ESCI, CCR-EXPANDED, IC

Timespan=All years

#10 TOPIC: ((depress* OR depressive* OR anxiety OR anxious OR anxi* OR mood OR emotion* OR sleep OR insomnia OR “sleep quality” OR “quality of life” OR QoL OR wellbeing OR well-being))

Indexes=SCI-EXPANDED, SSCI, A&HCI, CPCI-S, CPCI-SSH, BKCI-S, BKCI-SSH, ESCI, CCR-EXPANDED, IC

Timespan=All years

#9 TOPIC: ((“Mind-Body Therap*” OR “Mind Body Therap*”))

Indexes=SCI-EXPANDED, SSCI, A&HCI, CPCI-S, CPCI-SSH, BKCI-S, BKCI-SSH, ESCI, CCR-EXPANDED, IC

Timespan=All years

#8 TOPIC: ((“Tai-ji” OR “Tai Chi” OR “Chi, Tai” OR “Tai Ji Quan” OR “Ji Quan, Tai” OR “Quan, Tai Ji” OR Taiji OR Taijiquan OR “T'ai Chi” OR “Tai Chi Chuan” OR Qigong OR “Qi Gong” OR “Ch'i Kung” OR Baduanjin OR Wuqinxi OR Liuzijue OR “Yi Jin Jing”))

Indexes=SCI-EXPANDED, SSCI, A&HCI, CPCI-S, CPCI-SSH, BKCI-S, BKCI-SSH, ESCI, CCR-EXPANDED, IC

Timespan=All years

#7 TOPIC: ((acupuncture OR electroacupuncture OR pharmacopuncture OR moxibustion))

Indexes=SCI-EXPANDED, SSCI, A&HCI, CPCI-S, CPCI-SSH, BKCI-S, BKCI-SSH, ESCI, CCR-EXPANDED, IC

Timespan=All years

#6 TOPIC: ((acupressure OR tuina OR massage OR “massage therapy” OR “Chinese massage” OR “gua sha”))

Indexes=SCI-EXPANDED, SSCI, A&HCI, CPCI-S, CPCI-SSH, BKCI-S, BKCI-SSH, ESCI, CCR-EXPANDED, IC

Timespan=All years

#5 TOPIC: ((“traditional Chinese medicine” OR “Chinese medicine”))

Indexes=SCI-EXPANDED, SSCI, A&HCI, CPCI-S, CPCI-SSH, BKCI-S, BKCI-SSH, ESCI, CCR-EXPANDED, IC

Timespan=All years

#4 TOPIC: ((chemotherap* OR antineoplastic therap* OR cancer treatment*))

Indexes=SCI-EXPANDED, SSCI, A&HCI, CPCI-S, CPCI-SSH, BKCI-S, BKCI-SSH, ESCI, CCR-EXPANDED, IC

Timespan=All years

#3 TOPIC: ((cancer* OR neoplasm* OR tumor* OR tumour* OR malignan*))

Indexes=SCI-EXPANDED, SSCI, A&HCI, CPCI-S, CPCI-SSH, BKCI-S, BKCI-SSH, ESCI, CCR-EXPANDED, IC

Timespan=All years

#2 TOPIC: ((“cancer survivor*”))

Indexes=SCI-EXPANDED, SSCI, A&HCI, CPCI-S, CPCI-SSH, BKCI-S, BKCI-SSH, ESCI, CCR-EXPANDED, IC

Timespan=All years

#1 #3 OR #2

Indexes=SCI-EXPANDED, SSCI, A&HCI, CPCI-S, CPCI-SSH, BKCI-S, BKCI-SSH, ESCI, CCR-EXPANDED, IC

Timespan=All years

# Supplementary 2: Risk of Bias

| **Author** | **Bias arising from the randomization process** | **Bias due to deviations from intended intervention** | **Bias due to missing outcome data** | **Bias in measurement of the outcome** | **Bias in selection of the reported result** | **Overall** |
| --- | --- | --- | --- | --- | --- | --- |
| Qiao Xiao et al. (2025) | Low | Low | Low | Low | Low | Low |
| Lu et al. (2019) | Low | Low | Low | Low | Low | Low |
| Molassiotis et al. (2019) | Low | Low | Low | Some Concerns | Low | Some Concerns |
| Zhang et al. (2021) | Some Concerns | Low | Low | Low | Low | Some Concerns |
| Zhuang et al. (2024) | Low | Low | Low | Low | Low | Low |
| Xu et al. (2022) | Low | Low | Low | Low | Low | Low |
| Billhult et al. (2007) | Low | Low | Low | Low | Low | Low |
| Chuang et al. (2017) | Low | Low | Some Concerns | Low | Low | Some Concerns |
| Ding et al. (2020) | Some Concerns | Low | Some Concerns | Low | Low | Some Concerns |
| Wen et al. (2023) | Low | Low | Low | Low | Low | Low |
| Li et al. (2021) | Low | Low | Low | Low | Low | Low |
| Zhang et al. (2022) | Some Concerns | Low | Low | Low | Low | Some Concerns |
| Huang et al. (2015) | Low | Low | Low | Low | Low | Low |
| Huong et al. (2021) | High | High | High | Low | Low | High |
| Lin et al. (2019) | Low | High | High | Low | Low | High |
| Liu et al. (2022) | Low | Low | Low | Low | Low | Low |
| Molassiotis et al. (2014) | Some Concerns | Some Concerns | Some Concerns | Low | Low | Some Concerns |
| Yang et al. (2021) | Low | Some Concerns | Low | Low | Low | Some Concerns |
| Zhang et al. (2023) | Low | Low | Low | Low | Low | Low |
| Shin et al. (2016) | Some Concerns | Low | Low | Low | Low | Some Concerns |
| Tang et al. (2014) | High | Some Concerns | Some Concerns | Low | Low | High |
| Jung et al. (2023) | Low | Low | Some Concerns | Low | Low | Some Concerns |
| Yeh et al. (2016) | Low | Low | Low | Low | Low | Low |
| Zhang et al. (2016) | Low | Low | Low | Low | Low | Low |
| Zhou et al. (2017) | Low | Low | Low | Low | Low | Low |
| Chen et al. (2021) | Low | Some Concerns | Low | Low | Low | Some Concerns |
| Zhang et al. (2024) | Some Concerns | Low | High | High | Low | High |
| Zhou et al. (2022) | Some Concerns | Low | Some Concerns | Low | Low | Some Concerns |
| Zhang et al. (2020a) | Low | Low | Low | Low | Low | Low |
| Zhang et al. (2020b) | Low | Low | Low | Some Concerns | Low | Some Concerns |
| Peng et al. (2016) | Some Concerns | Low | Some Concerns | Low | Low | Some Concerns |
| Zhou et al. (2024) | Low | Low | Some Concerns | Low | Low | Some Concerns |
| Wu et al. (2022) | Low | Low | Some Concerns | Low | Low | Some Concerns |
| Wang et al. (2020) | Low | Low | Low | Low | Low | Low |
| Xiao et al. (2022) | Low | High | Low | Low | Low | High |

# Supplementary 3: Definitions and examples of TCM non-pharmacological intervention nodes

| **Full name** | **Definitions** | **Examples** |
| --- | --- | --- |
| **Acupuncture** | Acupuncture refers to a TCM intervention involving the insertion of fine needles into specific body acupoints along meridians, with the aim of regulating qi flow and restoring functional balance. In oncology-related supportive care, acupuncture is commonly used to alleviate chemotherapy-related symptoms such as fatigue, pain, sleep disturbance, anxiety, and gastrointestinal discomfort. Both manual acupuncture and electroacupuncture are included when needle insertion at body acupoints is the core therapeutic component. | Body acupuncture using standardized acupoints selected according to symptom profiles; manual acupuncture or electroacupuncture applied by licensed practitioners. |
| **Auricular Therapy** | Auricular Therapy encompasses auricular acupuncture or auricular acupressure based on the somatotopic representation of the body on the auricle. This modality stimulates specific auricular points to modulate central nervous system activity and autonomic regulation. Point selection typically follows internationally recognized auricular mapping systems, such as the WHO Standard Auricular Points Map. | Auricular acupressure or acupuncture targeting points such as Shenmen, Sympathetic, and Subcortex using press seeds or needles. |
| **Manual Acupoint Therapy** | Manual Acupoint Therapy refers to hands-on stimulation of body acupoints or meridian regions using tuina massage or body acupressure techniques. This modality applies manual pressure, kneading, or rolling to regulate meridian circulation and relieve symptom burden. It is commonly used for chemotherapy-related fatigue, sleep disturbance, and general discomfort. | Tuina massage or acupressure applied to body acupoints such as BL23 (Shenshu), BL15 (Xinshu), HT7 (Shenmen), and SP6 (Sanyinjiao). |
| **Mind–Body Exercise Therapy** | Mind–Body Exercise Therapy includes structured traditional exercises that integrate physical movement, breathing regulation, and mental focus to promote physical and psychological well-being. These practices emphasize coordinated posture, controlled respiration, and meditative awareness, and are widely used in cancer rehabilitation to improve quality of life, mood, sleep, and fatigue. | Tai Chi (e.g., 24-form), Qigong, Baduanjin, and other standardized traditional Chinese exercise forms. |
| **Moxibustion** | Moxibustion is a TCM intervention involving thermal stimulation of acupuncture points through the burning of moxa (Artemisia vulgaris). The heat generated is believed to warm meridians, promote circulation, and enhance systemic vitality. In supportive oncology care, moxibustion is commonly applied to relieve fatigue, pain, and digestive symptoms. | Indirect or direct moxibustion applied to body acupoints as specified in individual trials. |
| **CON (Control)** | Control conditions include routine care, usual supportive care, wait-list control, or no additional intervention beyond standard chemotherapy management. These controls do not involve active TCM non-pharmacological interventions. | Standard chemotherapy-related supportive care such as routine monitoring and health education without additional TCM intervention. |

**Reference**

1. World Health Organization. WHO International Standard Terminologies on Traditional Medicine in the Western Pacific Region. Geneva: WHO; 2007.
2. World Health Organization. WHO Standard Acupuncture Point Locations in the Western Pacific Region. Geneva: WHO; 2008.
3. MacPherson H, Altman DG, Hammerschlag R, et al. Revised STRICTA: standards for reporting interventions in clinical trials of acupuncture. PLoS Med. 2010;7(6):e1000261.

# Supplementary 4: Forest bias


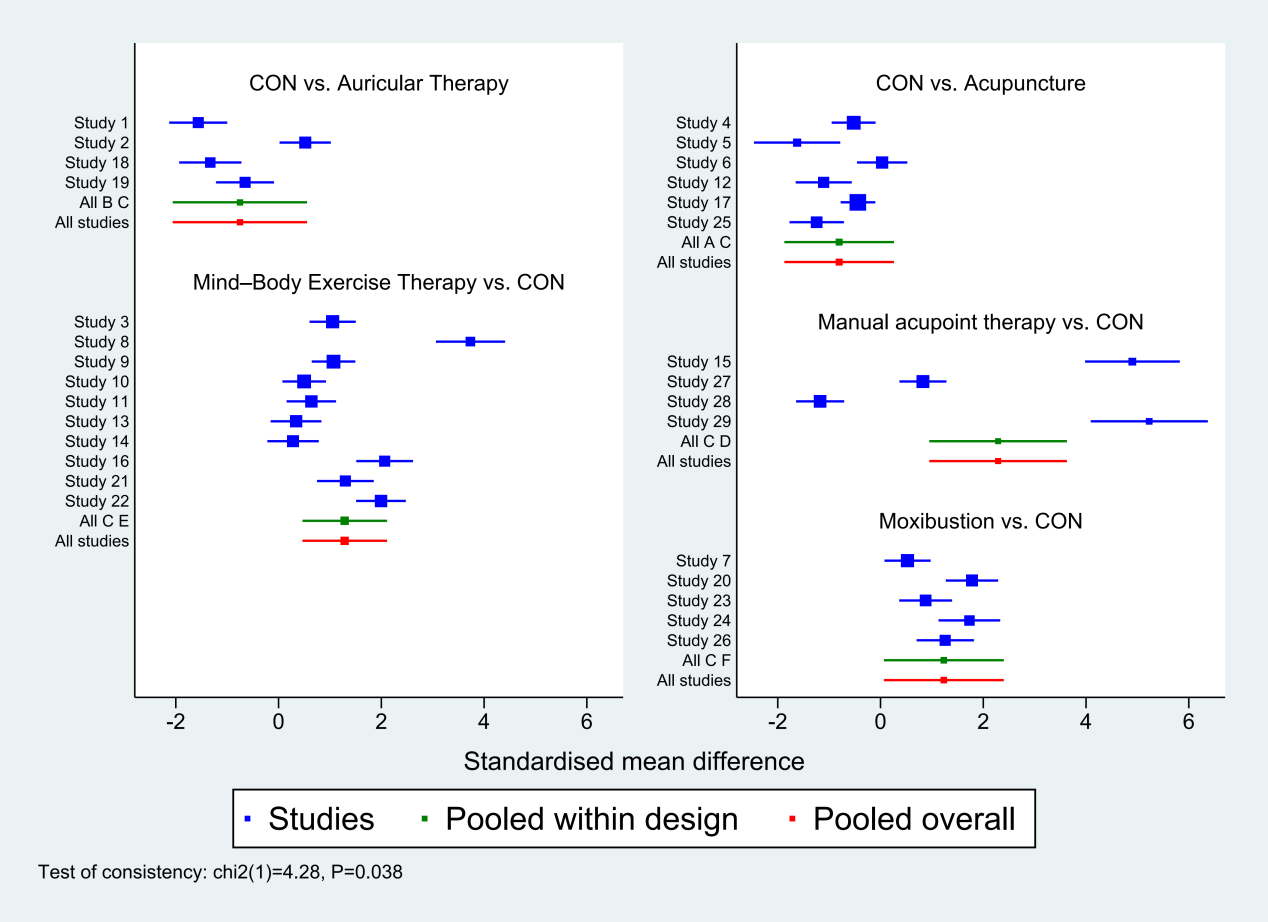


Figure 4.1 The forest plot of QoL. The result of inconsistency test showed the p=0.038.


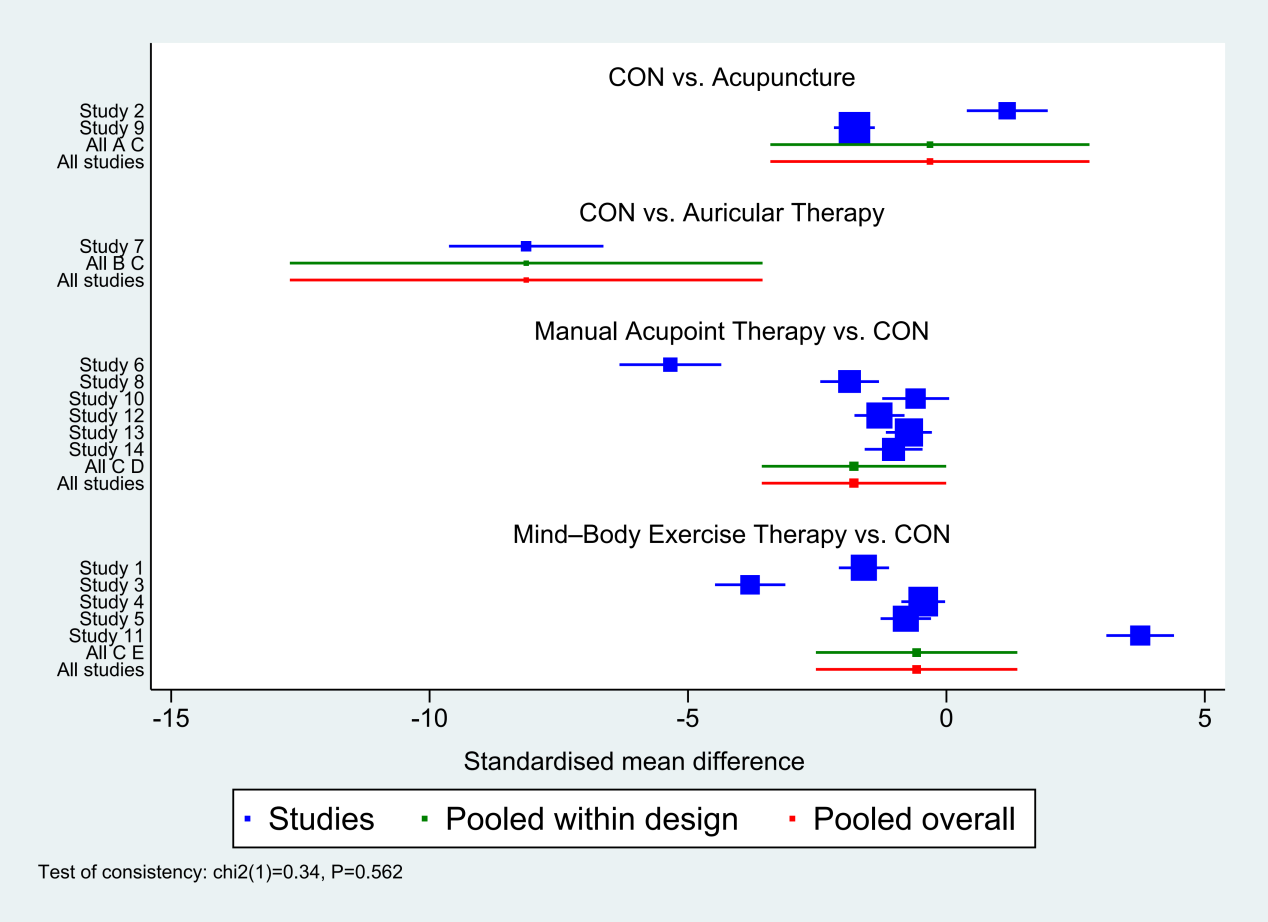


Figure 4.2 The forest plot of sleep quality. The result of inconsistency test showed the p=0.562.


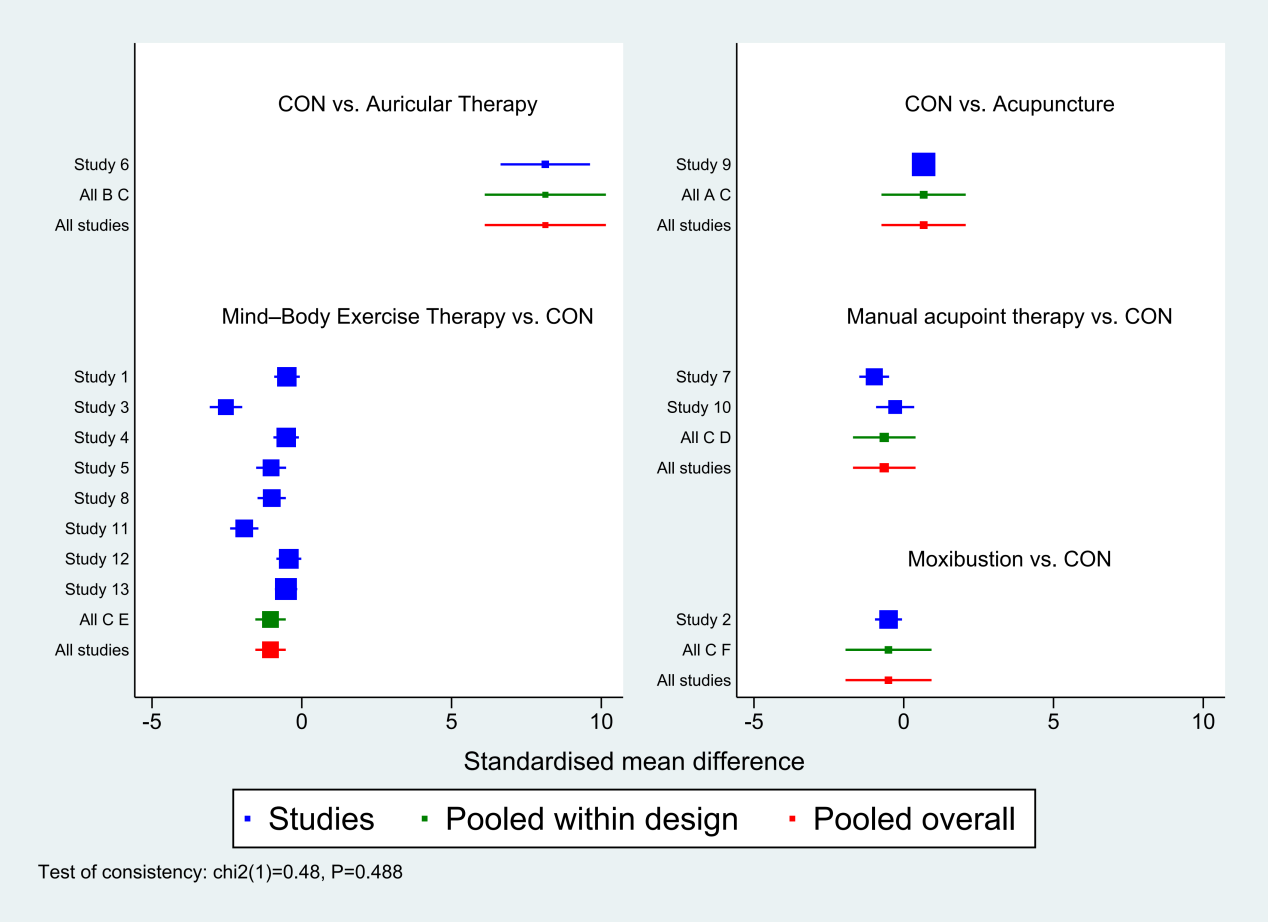


Figure 4.3 The forest plot of fatigue. The result of inconsistency test showed the p=0.488.


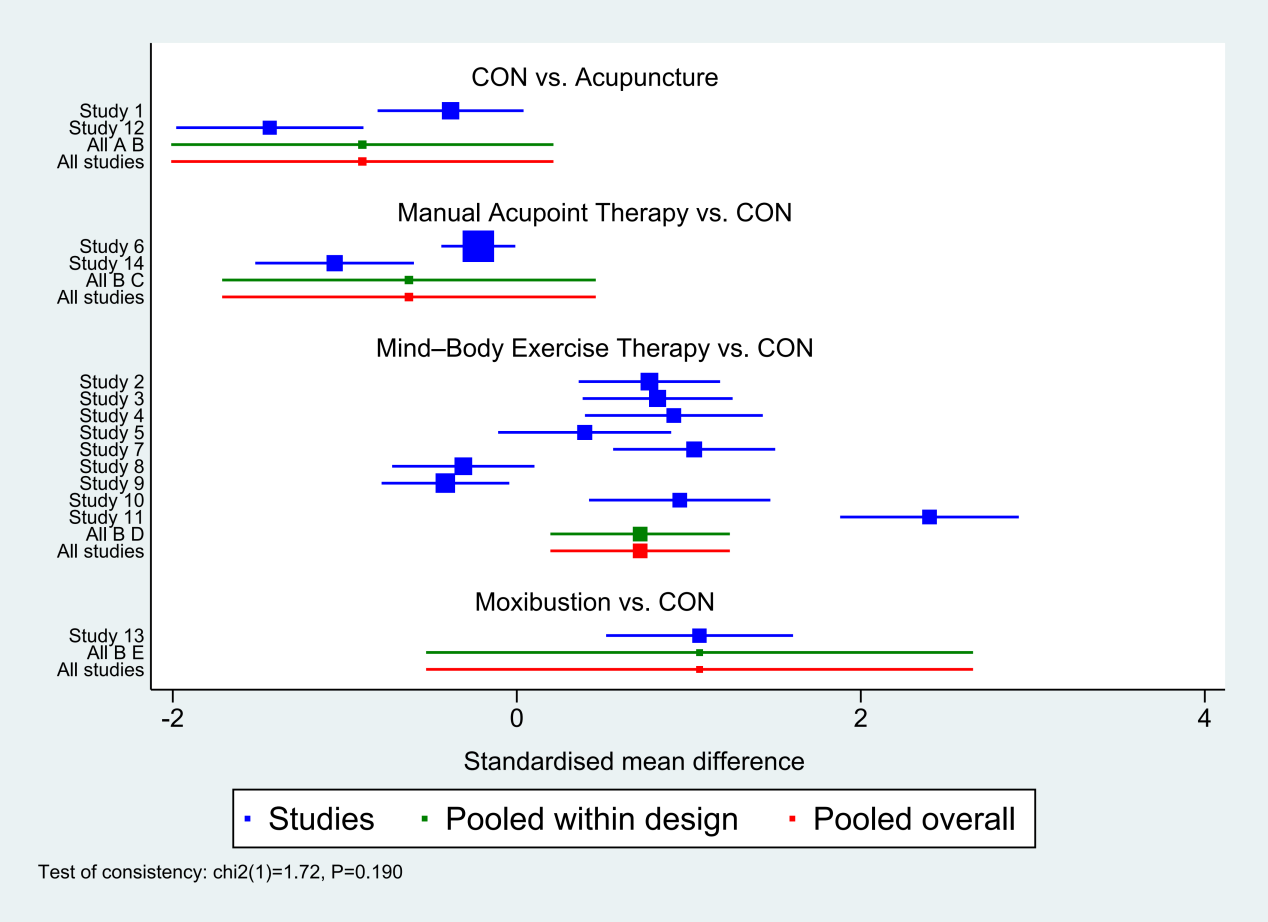


Figure 4.4 The forest plot of general mood. The result of inconsistency test showed the p=0.190.


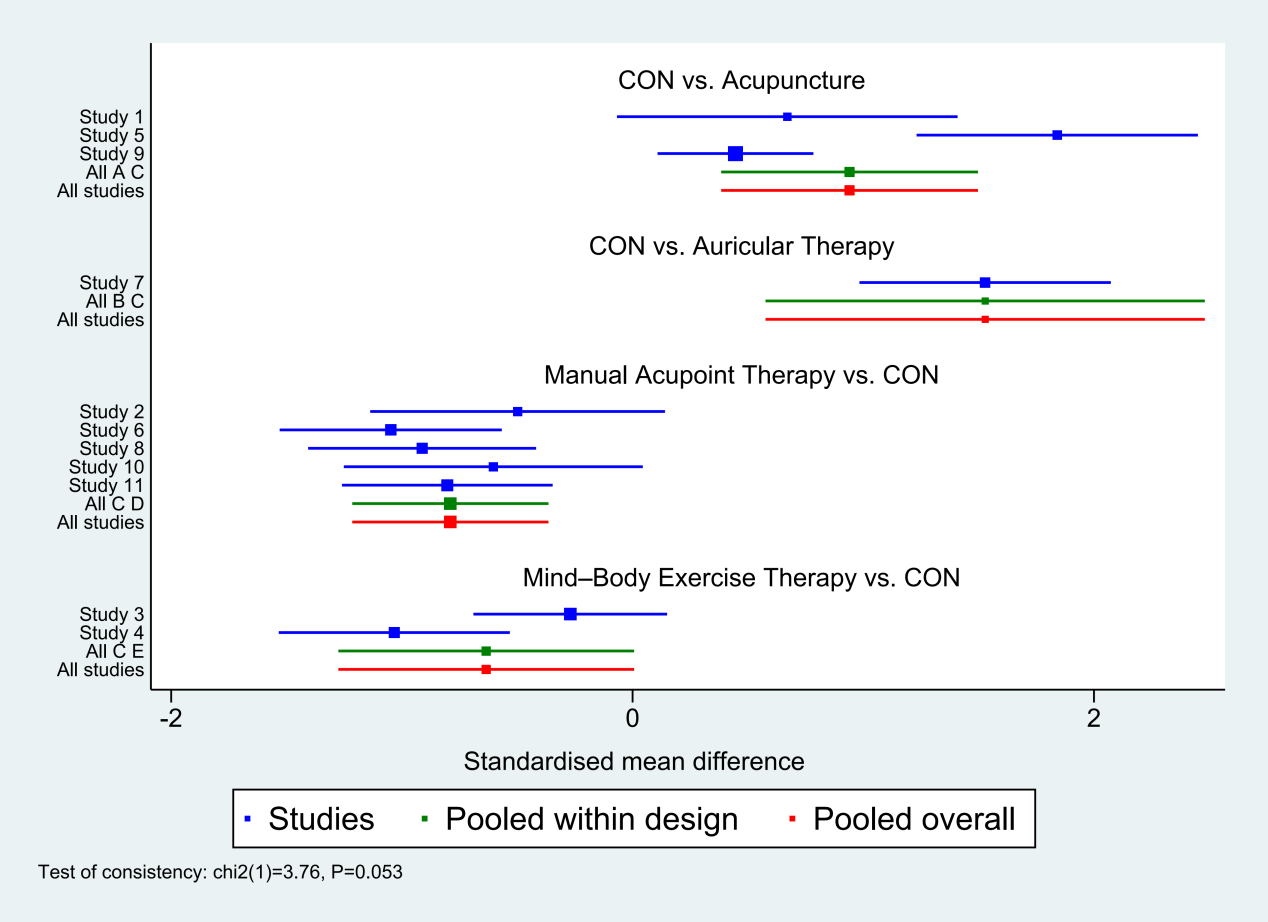


Figure 4.5 The forest plot of anxiety. The result of inconsistency test showed the p=0.053.


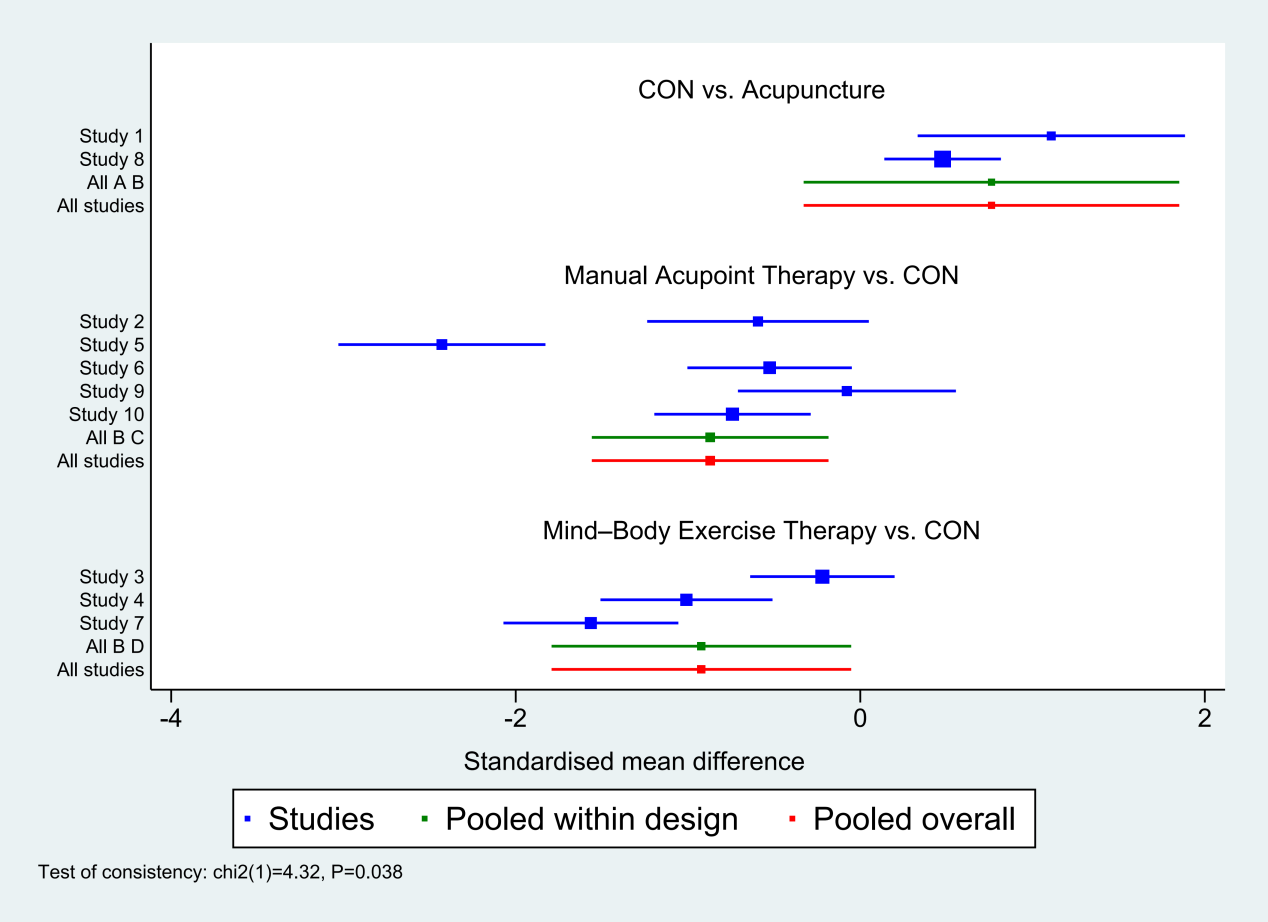


Figure 4.6 The forest plot of depression. The result of inconsistency test showed the p=0.038.

# Supplementary 5: Node splitting method results

Table 5.1 Results of the nodal splitting method for QoL

| **Side** | **Direct** |  | **Indirect** |  | **Difference** |  |  | **tau** |
| --- | --- | --- | --- | --- | --- | --- | --- | --- |
|  | **Coef.** | **Std. Err.** | **Coef.** | **Std. Err.** | **Coef.** | **Std. Err.** | **P>\|z\|** |  |
| A C * | -0.8055879 | 0.5438037 | -0.6791148 | 6.599522 | -0.1264731 | 6.621887 | 0.985 | 1.30254 |
| B C * | -0.7524973 | 0.6666636 | -1.613698 | 31.66165 | 0.8612008 | 31.66866 | 0.978 | 1.302373 |
| C D * | 2.28794 | 0.6827149 | 1.605464 | 31.66212 | 0.682476 | 31.66947 | 0.983 | 1.302378 |
| C E * | 1.288442 | 0.4199385 | 1.607883 | 20.04151 | -0.3194409 | 20.04591 | 0.987 | 1.302426 |
| C F * | 1.232195 | 0.5947897 | 1.608632 | 28.3232 | -0.376437 | 28.32945 | 0.989 | 1.302382 |

Table 5.2 Results of the nodal splitting method for depression

| **Side** | **Direct** |  | **Indirect** |  | **Difference** |  |  | **tau** |
| --- | --- | --- | --- | --- | --- | --- | --- | --- |
|  | **Coef.** | **Std. Err.** | **Coef.** | **Std. Err.** | **Coef.** | **Std. Err.** | **P>\|z\|** |  |
| A B * | 0.761491 | 0.5561822 | 0.4490018 | 11.18356 | 0.3124892 | 11.19739 | 0.978 | 0.7288735 |
| B C * | -0.8714363 | 0.3504629 | -1.514192 | 28.31301 | 0.6427553 | 28.3152 | 0.982 | 0.7288129 |
| B D * | -0.9222632 | 0.4435788 | -1.515684 | 36.54203 | 0.5934208 | 36.54472 | 0.987 | 0.7287731 |

# Supplementary 6: Characteristics of studies and subjects included in the review

Supplementary Table 6.1: Characteristics of included studies and participants

| **Study** | **Country/Region** | **Subjects  (intervention/ control)** | **Sex (male/female) (intervention/ control)** | **Mean age  (intervention/ control)** | **Type of disease** | **Intervention detail** | | **Treatment Duration** | **Outcomes** |
| --- | --- | --- | --- | --- | --- | --- | --- | --- | --- |
|  |  |  |  |  |  | **Intervention group** | **Control group** |  |  |
| Qiao Xiao et al. (2025) | China | 96 (32/32/32) | 23/9 vs. 22/10 vs. 19/13 | 64±9.1 vs. 59±10.2 vs. 64±13.3 | Gastric cancer, colon cancer, colorectal cancer | (a) Auricular point sticking (b) Ear scraping | Control (routine care): conventional prophylactic antiemetic regimen—IV tropisetron hydrochloride 5 mg in 100 mL 0.9% saline administered 30 min pre-chemotherapy, once daily for 3 days—plus routine nursing care (clinical monitoring, daily-living care, dietary guidance, health education, and emotional support). | From 1 day before chemotherapy to 5 days after chemotherapy | QoL |
| Lu et al. (2019) | China | 87 (43/44) | 26/17 vs. 30/14 | 55.60 ± 11.23 vs. 54.63 ± 11.88 | Colorectal cancer | Baduanjin Qigong | Control (routine care): standard care for cancer-related fatigue during chemotherapy, consisting of face-to-face health education on CRF (assessment methods, symptom recognition, contributing factors, and management strategies), delivered according to routine clinical practice; no structured exercise intervention was provided. | 24 weeks, ≥ 5 times/week, 20–40 minutes per session | QoL, Sleep quality, Fatigue |
| Molassiotis et al. (2019) | Hong Kong | 87 (44/43) | 9/35 vs. 15/28 | 57.1 ± 7.7 | Breast, colorectal, head and neck, gynecological cancers, multiple myeloma | Acupuncture | Control (routine care / standard care): standard oncology care for chemotherapy-induced peripheral neuropathy, consisting of usual medical management at the treating physician’s discretion (eg, analgesics, vitamin B12/B6 or other medications as needed), with no acupuncture or other complementary interventions during the study period (wait-list control). | 8 weeks, 2 times a week | QoL, General emotional state |
| Zhang et al. (2021) | Hong Kong | 30 (15/15) | 0/15 vs. 0/15 | 52.5 ± 8.9 vs. 52.7 ± 6.3 | Breast cancer | Electroacupuncture + Auricular Acupressure | Control (routine care / wait-list control): standard oncologic symptom management provided by treating oncologists (ie, usual care for chemotherapy-associated symptoms, including insomnia management as clinically indicated), with no acupuncture or auricular acupressure during the 6-week waiting period; participants continued routine medical care and received the acupuncture intervention after the waiting phase | 6 weeks, 2 times per week, 12 times in total | QoL, Sleep quality, Anxiety, Depression |
| Zhuang et al. (2024) | China | 64 (33/31) | 16/17 vs. 12/19 | 57.5 ± 9.8 vs. 55.5 ± 10.6 | Various malignant tumors (such as breast cancer, lung cancer, stomach cancer, intestinal cancer, ovarian cancer, etc.) | Ginger moxibustion + acupuncture | Control (routine care): standard antiemetic care consisting of oral ondansetron hydrochloride 8 mg, administered three times daily for 3 consecutive days during chemotherapy, in accordance with routine clinical practice; no additional nonpharmacologic interventions were provided. | 3 days, during chemotherapy | QoL |
| Xu et al. (2022) | China | 79 (40/39) | 20/20 vs. 18/21 | 56.62 ± 8.36 vs. 56.25 ± 9.05 | Non-small-cell lung cancer | Mild moxibustion | Control (routine care): standard oncology care consisting of guideline-based GP chemotherapy plus routine nursing care, including chemotherapy administration and monitoring, prophylactic antiemetic and gastric-protective medications as needed, hydration and renal protection, catheter care, dietary guidance, emotional support, exercise advice, and routine follow-up; no moxibustion intervention was provided. | 30 minutes a day for 12 days | QoL, Fatigue |
| Billhult et al. (2007) | Sweden | 39 (19/20) | 0/19 vs. 0/20 | 50.5 ± 10.1 vs. 53.1 ± 8.4 | Breast cancer | Effleurage massage | Control (routine care): standard oncology care during chemotherapy, including routine clinical monitoring and guideline-based antiemetic prophylaxis (5-HT3 receptor antagonist tropisetron plus corticosteroid), with five 20-min staff visits consisting of unstructured conversation and no massage or additional nonpharmacologic intervention. | 5 sessions during chemotherapy sessions 3–7 | Anxiety, Depression |
| Chuang et al. (2017) | Taiwan | 96 (48/48) | 26/22 vs. 29/19 | 55.85 ± 16.78 vs. 64.54 ± 15.51 | Non-Hodgkin lymphoma | Chan-Chuang Qigong | Control (routine care): conventional care during chemotherapy, consisting of usual medical and nursing care per institutional practice, including standard chemotherapy administration and symptom management, with provision of a nursing guidance booklet on chemotherapy-related side effects (eg, nausea, vomiting, fatigue); no qigong or structured exercise intervention was provided. | 2-3 times a day for 21 days | QoL, Sleep quality, Fatigue |
| Ding et al. (2020) | China | 98（49/49） | 21/28 vs. 24/25 | 53.20 ± 6.80 / 50.80 ± 7.60 | Malignant tumors receiving long-term chemotherapy with PICC | 24-posture simplified Tai Chi | Control (routine care): standard PICC home-care education provided pre-discharge by specialized PICC nurses (catheter function demonstration, PICC maintenance manual, precautions and importance of catheter care), followed by a 3-month home-based handgrip (grip-ball) exercise program (10-s squeeze/10-s rest repeated ×20 per set; one set each morning/noon/evening) with instruction before discharge and regular follow-up/monitoring; no tai chi was provided. | Exercise ≥ 5 times a week, about 60 minutes each time, for 3 months | QoL, General emotional state |
| Wen et al. (2023) | China | 88 (44/44) | 34/10 vs. 33/11 | 45.55 ± 8.99 / 47.07 ± 9.43 | Nasopharyngeal carcinoma | Baduanjin Qigong | Control (routine care/usual care): conventional post-chemoradiotherapy care, including routine health education, clinical condition monitoring, and psychological care; upon discharge, patients received standard oncologist-led instructions on diet, exercise/rehabilitation exercise, and follow-up/review. Participants were advised to maintain usual lifestyle/physical activity (with exercise at a tolerable intensity) and received no Baduanjin intervention during the 12-week study period. | 12 weeks, 5 days a week, 40 minutes per session | QoL, Sleep quality, General emotional state, Anxiety, Depression, Fatigue |
| Li et al. (2021) | China | 70 (35/35) | 0/35 vs. 0/35 | 48.46 ± 8.66 / 49.77 ± 8.17 | Breast cancer | Baduanjin Qigong | Control (routine care/usual care): Standard health education/usual lifestyle advice delivered via a brochure-based, face-to-face counseling session; participants were instructed to maintain their usual healthy lifestyle, with disease-related queries addressed ad hoc by study staff via WeChat or in-person/oral communication. | 12 weeks, 5 days a week, 45 minutes per session | QoL, Sleep quality, Anxiety, Depression, Fatigue |
| Zhang et al. (2022) | China | 60 (30/30) | NA | NA | Breast cancer | Acupuncture and Moxibustion | Control (routine care): standard chemotherapy (adriamycin + cisplatin) with routine prophylactic antiemetic therapy, specifically IV tropisetron hydrochloride administered 1 h before chemotherapy, for 5 days; no ginger-partitioned moxibustion was provided. | Twice a week for 3 chemotherapy cycles (21 days per cycle) | QoL, Anxiety |
| Huang et al. (2015) | Taiwan | 95 (33/31/31) | 0/33 vs. 0/31 vs. 0/31 | 51.4 | Breast cancer | (a) Qigong (b) Taichi | Control (routine care/usual care): guideline-based standard oncology care during chemotherapy plus a standardized post-surgical rehabilitation exercise program developed by hospital clinicians (e.g., finger/palm and shoulder range-of-motion and strengthening exercises such as wall climbing, pulley/towel/rod exercises, chest flies, and functional upper-limb tasks); home-based, 30 min/session, 3 sessions/week for 12 weeks, with at least one training session, DVD instruction, and weekly telephone follow-up; no qigong intervention | 3 times/week, 30 minutes each time, for 12 weeks | QoL, General emotional state |
| Huong et al. (2021) | Vietnam | 76 (38/38) | 9/29 vs.15/23 | 54.6 ± 10.9 vs. 56.6 ± 9.0 | Multiple cancer types (breast cancer is the most common, followed by lung cancer, digestive tract cancer, cervical cancer, lymphoma, etc.) | Acupressure | Control (routine care / enhanced standard care): standard oncology care during chemotherapy, supplemented with a 30-min structured education session providing general recommendations for managing insomnia, anxiety, and depression (e.g., sleep hygiene, lifestyle and coping advice), plus weekly brief telephone follow-up with neutral conversation; participants had access to usual medical and nursing care as clinically indicated, with no acupressure intervention. | Once a day for 4 weeks; total follow-up 8 weeks | QoL, Sleep quality, Anxiety, Depression |
| Lin et al. (2019) | China | 68 (34/34) | 18/16 vs. 23/11 | 59.50±9.21 vs. 61.85±8.48 | Non-small cell lung cancer | Auricular acupressure | Control (routine care): usual care during hospitalisation and after discharge, including routine clinical monitoring and supportive follow-up (regular telephone follow-up and home visits) per institutional practice; no auricular acupressure intervention was delivered (participants could opt to receive auricular acupressure after the 9-week follow-up, if desired). |  | Sleep quality, Anxiety, Fatigue |
| Liu et al. (2022) | China | 70 (36/34) | 0/36 vs. 0/34 | 51.4 ± 8.85 | Breast cancer | Acupressure | Control (routine care/usual care): routine inpatient nursing care in the breast oncology department, including symptom assessment and management, diet counseling, rest and exercise guidance, and usual psychological support; no MBSR or acupressure was provided. | 8 weeks | Sleep quality, Anxiety, Depression, Fatigue |
| Molassiotis et al. (2014) | United Kingdom | 334 (168/166) | 39/129 vs. 38/128 | >50 years | Breast cancer, colorectal cancer, gynecologic cancer, lung cancer | Acupressure | Control (routine care / standard care): guideline-based standardized antiemetic prophylaxis per ASCO/MASCC recommendations (NK1 antagonists not routinely used), with rescue antiemetics permitted for persistent/severe nausea or vomiting at the treating clinician’s discretion; no acupressure wristbands were provided. | Each chemotherapy cycle lasts 7 days, for a total of 4 cycles | General emotional state |
| Yang et al. (2021) | China | 79 (40/39) | 24/16 vs. 23/17 | 18–75 | Gastric or colorectal cancer | Five Animal Exercises Qigong | Control (routine care): conventional therapy during chemotherapy, comprising coping strategies to prevent/mitigate chemotherapy-related adverse reactions, routine patient education (individualized information on disease course and treatment, expected symptoms, and supportive nutrition), and emotional support primarily from family and clinical staff; standard psychosocial care (e.g., cognitive therapy and stress management) was also provided. | 4 weeks | QoL, General emotional state, Depression, Fatigue |
| Zhang et al. (2023) | Hong Kong | 138 (69/69) | 0/69 vs. 0/69 | 51.7 ± 9.6 / 52.7 ± 8.3 | Breast cancer | Acupuncture + auricular acupressure | Control (routine care / sham control): standard oncology care and routine symptom management continued throughout the study, with psychotropic medications (e.g., sedatives/hypnotics/anxiolytics) permitted as clinically indicated; participants received sham acupuncture (non-penetrating needling at non-acupoints plus sham auricular acupressure) and no active acupuncture. | 6 weeks of intensive treatment + 12 weeks of maintenance treatment | QoL, Sleep quality, Anxiety, Depression, Fatigue |
| Shin et al. (2016) | South Korea | 52 (26 / 26) | 0/26 vs. 0/26 | NA | Breast cancer | Auricular acupressure | Control (routine care/usual care): standard oncology care during chemotherapy, comprising usual medical and nursing management per institutional practice, including routine symptom monitoring and supportive care (e.g., diet and activity guidance and medications such as antiemetics, analgesics, laxatives as clinically indicated); no auricular acupressure or sham intervention was provided. | 6 weeks | QoL |
| Tang et al. (2014) | Taiwan | 40 (24/16) | 12/12 vs. 12/4 | 54.8 ± 9.5 vs. 66.1 ± 8.0 | Lung cancer | Acupressure | Control (routine care / sham control): standard oncology care during chemotherapy, including routine clinical monitoring and supportive care per institutional practice, plus sham acupressure applied at non-acupoint locations with the same frequency and duration as the intervention; no therapeutic acupressure or essential oils were provided. | 5 months | Sleep quality, Anxiety, Depression, Fatigue |
| Jung et al. (2023) | South Korea | 51 (25/26) | 0/25 vs. 0/26 | 49.24 ± 7.57 / 49.73 ± 5.19 | Breast cancer | Auricular acupressure | Control (routine care / sham control): standard oncology care for chemotherapy-induced peripheral neuropathy continued throughout the study, including routine clinical monitoring and usual supportive/symptom management as clinically indicated; participants additionally received sham auricular acupressure (Vaccaria seeds applied to non-therapeutic ear points unrelated to CIPN, with the same schedule and procedures as the intervention), with no active auricular acupressure. | 3 weeks | QoL |
| Yeh et al. (2016) | Taiwan | 102 (51/51) | 28/23 vs. 29/22 | 59.79 ± 16.54 | Non-Hodgkin’s lymphoma | Chan-Chuang Qigong | Control (routine care/usual care): standard oncology care during chemotherapy per institutional practice, without any structured exercise intervention, including routine clinical monitoring and usual supportive care as clinically indicated (e.g., symptom management and nursing support). | 21 days | Sleep quality, Fatigue |
| Zhang et al. (2016) | China | 91 (47 / 44) | 37/10 vs. 31/13 | 62.8 years | Lung cancer | Taichi | Control (routine care / active control): standard oncology care during chemotherapy per institutional practice, plus a low-impact exercise program consisting of gentle arm, neck, and leg movements, stretching of upper and lower body muscle groups, and deep abdominal breathing. Exercises were performed every other day for 60 min over 12 weeks, with no Tai Chi intervention. | 12 weeks | General emotional state, Fatigue |
| Zhou et al. (2017) | China | 114 (57/57) | 38/19 vs. 45/12 | NA | Nasopharyngeal carcinoma | Taichi | Control (routine care/usual care): standard care for nasopharyngeal carcinoma during chemoradiotherapy per institutional practice, including routine medical and nursing management and symptom monitoring; no structured exercise or Tai Chi intervention was provided. | 7 weeks | General emotional state, Fatigue |
| Chen et al. (2021) | China | 84 (42/42) | 25/17 vs. 26/16 | 57.93 ± 6.77 vs. 57.62 ± 6.94 | Lung cancer | Moxibustion + Acupoint Therapy | Control (routine care): routine oncology specialty nursing during chemotherapy, including maintenance of chemotherapy access, medication guidance, health education, psychological counseling, and nutritional support; no moxibustion, auricular acupressure, or acupoint application was provided. | 6 weeks | QoL |
| Zhang et al. (2024) | China | 62 (31/31) | 20/11 vs. 19/12 | 46.53 ± 4.12 vs. 47.36 ± 3.27 | Rectal cancer | Baduanjin Qigong | Control (routine care/usual care): routine nursing care during neoadjuvant chemoradiotherapy, delivered per institutional practice, including physician-ordered treatment-related nursing care, psychological counseling, and dietary guidance; no Baduanjin exercise or traditional Chinese medicine–based emotional care was provided. | 1 year | QoL, General emotional state |
| Zhou et al. (2022) | China | 100 (50/50) | 16/34 vs. 18/32 | 55 ± 8.5 vs 54 ± 7.75 | Gastrointestinal tumors | Baduanjin Qigong | Control (routine care/usual care): routine nursing care during post-chemotherapy hospitalization, delivered per institutional practice, including physician-prescribed chemotherapy-related nursing care, medication administration, dietary guidance, psychological counseling, rest and activity advice, and discharge education; no Baduanjin exercise or traditional Chinese medicine–based emotional nursing was provided. | 4 weeks | QoL, General emotional state |
| Zhang et al. (2020a) | China | 64 (32/32) | 18/14 vs. 17/15 | 33 ± 5 | Advanced malignant bone tumors | Ginger moxibustion | Control (routine care): standard chemotherapy (adriamycin + cisplatin) with guideline-based prophylactic antiemetic therapy, specifically IV tropisetron hydrochloride 5 mg administered 1 h before chemotherapy, once daily for 5 days; routine oncology care otherwise continued, with no ginger-partitioned moxibustion | 5 days | QoL |
| Zhang et al. (2020b) | China | 60 (30/30) | 16/14 vs. 15/15 | 51.1 ± 4.7 vs. 52.2 ± 5.1 | Lung cancer | Ginger moxibustion | Control (routine care): standard chemotherapy with guideline-based prophylactic antiemetic therapy, consisting of IV tropisetron (5 mg in 20 mL 0.9% saline) administered 1 h before chemotherapy, once daily for 3 days, alongside usual oncology care and symptom monitoring; no ginger-partitioned moxibustion was provided. | 3 days | QoL |
| Peng et al. (2016) | China | 66 (33/33) | 27/6 vs. 24/9 | 46.67 ± 12.03 vs. 49.21 ± 13.44 | Nasopharyngeal carcinoma | Acupuncture | Control (routine care): guideline-based standard concurrent radiotherapy and chemotherapy for stage III–IVa nasopharyngeal carcinoma per institutional protocols, including routine medical and nursing management and symptom monitoring; no pulse acupuncture or other adjunctive acupuncture interventions were provided | 2 months | QoL, General emotional state |
| Zhou et al. (2024) | China | 60 (30/30) | 19/11 vs. 21/9 | 59 ± 11 vs. 61 ± 11 | Various cancers undergoing chemotherapy with platinum-based drugs | Thermal moxibustion | Control (routine care): guideline-based standard antiemetic care for chemotherapy-induced nausea and vomiting, consisting of tropisetron hydrochloride administered per national expert consensus—IV tropisetron 5 mg in 100 mL 0.9% saline 30 min before chemotherapy on day 1, followed by oral tropisetron 5 mg once daily on days 2–7—with routine oncology care and symptom monitoring; no heat-sensitive moxibustion was provided. | 7 days | QoL, General emotional state |
| Wu et al. (2022) | China | 80 (40/40) | 23/17 vs. 24/16 | 60.12 ± 3.88 vs. 60.09 ± 3.37 | Various cancers undergoing chemotherapy with platinum-based drugs | Acupressure + plaster | Control (routine care/usual care): routine nursing care during chemotherapy, including ward environment management, psychological support and patient education, and dietary guidance to ensure adequate nutrition; no traditional Chinese medicine nursing, herbal patch application, or acupoint massage was provided. | 1 week | QoL, Sleep quality, Anxiety, Depression |
| Wang et al. (2020) | China | 83 (41/42) | 23/18 vs. 24/18 | 50.52 ± 4.05 vs. 50.11 ± 3.94 | Lung cancer | Acupressure | Control (routine care/usual care): routine nursing care during chemotherapy, including pre-chemotherapy health education, medication-assisted management of chemotherapy-related adverse effects, dietary guidance, and oral care; additional supportive measures included sleep-hygiene advice (e.g., warm foot baths before bedtime, quiet ward environment). No traditional Chinese medicine–based emotional nursing or acupoint massage was provided. | 1-2 weeks | QoL, Sleep quality, General emotional state |
| Xiao et al. (2022) | China | 56 (28/28) | 16/12 vs. 17/11 | 58.48 ± 4.25 vs. 58.51 ± 4.18 | Various malignant tumors undergoing chemotherapy | Acupressure + plaster | Control (routine care/usual care): standard chemotherapy-related pharmacologic management and routine supportive nursing for gastrointestinal reactions, including prophylactic antiemetic therapy (IV tropisetron hydrochloride 5 mg in 100 mL solution once daily before chemotherapy), symptom-directed medications (e.g., metoclopramide, ondansetron, sedatives as needed), dietary guidance, hydration and electrolyte management, oral care, and routine psychological support; no acupoint massage or acupoint application was provided. | 7 days | QoL, Sleep quality |

Note: PICC, Peripherally Inserted Central Catheter. QoL, Quality of Life. NA, Not Available.

Supplementary Table 6.2: Extracted post-intervention mean ± standard deviation values for outcome measures across study arms

| **Study** | **QoL** | | | **Sleep Quality** | | | **Fatigue** | | | **General Mood** | | | **Anxiety** | | | **Depression** | | |
| --- | --- | --- | --- | --- | --- | --- | --- | --- | --- | --- | --- | --- | --- | --- | --- | --- | --- | --- |
|  |  |  |  |  |  |  |  |  |  |  |  |  |  |  |  |  |  |  |
| Qiao Xiao et al. (2025) |  | (a) 24h：83 ± 13.14 5d：96.5 ± 8.88 (b) 24h：97 ± 12.78，5d：92 ± 13.33 | 24h：78 ± 3.33 5d：78 ± 2.96 |  |  |  |  |  |  |  |  |  |  |  |  |  |  |  |
| Lu et al. (2019) | KPS | pre: 76.5 ± 11.3 post: 89.3 ± 8.3 | pre: 74.8 ± 14.1 post: 75.2 ± 11.5 | PSQI | pre: 10.5 ± 1.5 post: 4.1 ± 1.1 | pre: 10.5 ± 2.1 post: 6.9 ± 2.0 | BFI | pre: 4.4 ± 2.2 post: 2.7 ± 2.1 | pre: 4.7 ± 2.5 post: 4.1 ± 1.9 |  |  |  |  |  |  |  |  |  |
| Molassiotis et al. (2019) | FACT-G | pre: 71.5±11.9 post: 76.7±11.1 | pre: 72.9±15.9 post: 70.7±16.4 |  |  |  |  |  |  | Symptom Distress Scale | pre: 16.6±4.6 post: 14.6±3.9 | pre: 17.6±5.9 post: 17.6±5.9 |  |  |  |  |  |  |
| Zhang et al. (2021) | FACT-B | change: 14.9 ± 8.3 | change: 1.3 ± 8 | PSQI | change: −3.9 ±1.9 | change: -1.6 ±1.9 |  |  |  |  |  |  | HADS-Anxiety | change: -1.4 ±1.5 | change: -0.4 ±1.4 | HADS-Depression | change: -2.2 ±1.5 | change: -0.6 ±1.3 |
| Zhuang et al. (2024) | KPS | pre: 62.64 ± 3.21 post: 59.06 ± 4.05 | pre: 61.94 ± 3.06 post: 58.48 ± 3.90 |  |  |  |  |  |  |  |  |  |  |  |  |  |  |  |
| Tan et al. (2022) |  |  |  |  |  |  |  |  |  |  |  |  |  |  |  |  |  |  |
| Xu et al. (2022) | QLQ-CCC | pre: 125.20 ± 4.01 8days: 119.87 ± 5.52 21days: 118.66 ± 3.87 | pre: 126.01 ± 6.37 8days: 117.80 ± 4.17 21days: 116.92 ± 2.66 |  |  |  | RPFS-CV | pre: 5.31 ± 0.48 8days: 6.28 ± 0.35 21days: 6.44 ± 0.38 | pre: 5.35 ± 0.55 8days: 6.60 ± 0.32 21days: 6.72 ± 0.41 |  |  |  |  |  |  |  |  |  |
| Billhult et al. (2007) |  |  |  |  |  |  |  |  |  |  |  |  | HADS Anxiety | change:−0.1 ± 2.9 | change: +1.3 ± 2.6 | HADS Depression | change:−0.7 ± 2.8 | change: +0.6 ± 1.22 |
| Chuang et al. (2017) | EORTC QLQ-C30 | pre: 35.07 ± 10.45 post: 80.56 ± 14.92 | pre: 34.94 ± 9.84 post: 36.18 ± 10.17 | VSHSS | change: +265.83 ± 94.56 | change: +0.21 ± 25.75 | BFI-TF | pre: 5.49 ± 1.02 post: 0.37 ± 1.39 | pre: 6.55 ± 1.42 post: 4.95 ± 1.57 |  |  |  |  |  |  |  |  |  |
| Ding et al. (2020) | SF-36 | pre: 53.82 ± 14.82 post: 72.50 ± 12.50 | pre: 50.37 ± 13.84 post: 53.74 ± 15.20 |  |  |  |  |  |  | SF-36 | pre: 68.09 ± 10.77 post: 78.77 ± 10.29 | pre: 65.82 ± 9.76 post: 68.41 ± 10.73 |  |  |  |  |  |  |
| Wen et al. (2023) | FACT-H&N | pre: 72.67 ± 14.81 post: 101.08 ± 12.20 | pre: 68.44 ± 16.99 post: 88.87 ± 18.39 | PSQI | pre: 8.64 ± 3.76 post: 4.58 ± 3.53 | pre: 9.61 ± 3.67 post: 7.33 ± 4.57 | MFI-20 | pre: 62.89 ± 8.39 post: 48.08 ± 10.52 | pre: 64.56 ± 9.22 post: 54.95 ± 10.82 | Emotional well-being | pre: 10.33 ± 2.22 post: 16.03 ± 1.56 | pre: 9.95 ± 1.76 post: 14.08 ± 1.88 | GAD-7 | pre: 5.80 ± 2.12 post: 3.64 ± 1.08 | pre: 6.09 ± 2.33 post: 4.46 ± 1.51 | PHQ-9 | pre: 7.00 ± 2.43 post: 4.70 ± 1.41 | pre: 7.27 ± 2.41 post: 5.46 ± 2.17 |
| Li et al. (2021) | FACT-B | pre: 87.14 ± 11.40 post: 101.86 ± 10.79 | pre: 85.74 ± 10.86 post: 93.34 ± 11.12 | PSQI | pre: 10.57 ± 3.05 post: 6.54 ± 2.49 | pre: 10.54 ± 2.79 post: 8.71 ± 2.65 | CFS | pre: 40.40 ± 5.77 post: 30.43 ± 5.02 | pre: 39.83 ± 6.09 post: 35.86 ± 6.19 |  |  |  | SAS | pre: 52.06 ± 5.43 post: 44.20 ± 4.95 | pre: 52.51 ± 4.84 post: 49.83 ± 4.54 | SDS | pre: 51.57 ± 6.38 post: 42.66 ± 4.37 | pre: 51.40 ± 5.91 post: 48.23 ± 5.23 |
| Zhang et al. (2022) | WHOQOL-BREF | pre: 65.54 ± 8.66 post: 81.47 ± 8.25 | pre: 65.33 ± 8.23 post: 72.11 ± 7.45 |  |  |  |  |  |  |  |  |  | HAMA | pre: 18.65 ± 3.06 post: 10.49 ± 1.85 | pre: 18.73 ± 3.02 post: 15.61 ± 2.31 |  |  |  |
| Huang et al. (2015) | SF-36 | (a) pre: 46.4 ± 6.3 1 month: 47.1 ± 6.6 3 month: 47.2 ± 6.0  (b) pre: 45.3 ± 7.5 1 month: 42.6 ± 7.0 3 month: 45.8 ± 7.5 | pre: 46.7 ± 10.4 1 month: 44.7 ± 7.2 3 month: 44.7 ± 9.1 |  |  |  |  |  |  | SF-36  Mental Component Score | (a) pre: 28.1 ± 12.4 1 month: 30.7 ± 15.0 3 month: 36.2 ± 14.8  (b) pre: 39.5 ± 14.1 1 month: 39.2 ± 12.7 3 month: 40.3 ± 13.0 | pre: 38.0 ± 12.7 1 month: 35.2 ± 12.0 3 month: 33.3 ± 14.9 |  |  |  |  |  |  |
| Huong et al. (2021) | FACT-G | pre: 52.56 ± 2.44 4 weeks: 61.25 ± 3.09 8 weeks: 59.46 ± 3.12 | pre: 66.00 ± 1.90 4 weeks: 61.90 ± 2.58 8 weeks: 61.49 ± 3.33 | ISI | pre: 20.2 ± 0.62 8 weeks: 14.71 ± 1.34 | pre: 20.26 ± 0.52 8 weeks: 19.76 ± 0.98 |  |  |  |  |  |  | HADS-A | pre: 12.13 ± 0.6 8 weeks: 9.26 ± 0.91 | pre: 11.86 ± 0.61 8 weeks: 10.19 ± 0.85 | HADS-D | pre: 11.00 ± 0.48 4 weeks: 9.58 ± 0.79 8 weeks: 9.30 ± 0.80 | pre: 10.23 ± 0.43 4 weeks: 10.56 ± 0.85 8 weeks: 11.35 ± 0.88 |
| Lin et al. (2019) |  |  |  | PSQI | pre: 9.59 ± 2.09 post: 8.27 ± NA | pre: 26.29 ± 3.12 post: 9.64 ± NA | CFS | pre: 27.18 ± 2.84 post: 20.29 ± NA | pre: 26.29 ± 3.12 post: 26.49 ± NA |  |  |  | SAS | pre: 49.65 ± 3.39 post: 40.62 ± NA | pre: 47.74 ± 3.96 post: 44.41 ± NA |  |  |  |
| Liu et al. (2022) |  |  |  | PSQI | pre: 11.42 ± 2.38 post: 7.03 ± 2.15 | pre: 11.15 ± 1.73 post: 11.26 ± 2.84 | BFI-C | pre: 6.79 ± 1.10 post: 5.25 ± 1.36 | pre: 7.00 ± 2.01 post: 7.01 ± 1.54 |  |  |  | HADS-A | pre: 11.25 ± 3.07 post: 9.06 ± 3.07 | pre: 11.18 ± 2.12 post: 11.47 ± 2.29 | HADS-D | pre: 10.69 ± 3.38 post: 10.17 ± 3.46 | pre: 10.29 ± 2.26 post: 11.44 ± 3.14 |
| Molassiotis et al. (2014) |  |  |  |  |  |  |  |  |  | SCL | pre: 45.9 ± 26.1 post: 29.7 ± 21.1 follow-up: 33.1 ± 27.4 | pre: 42.7 ± 23.6 post: 21.4 ± 18.2 follow-up: 19.7 ± 14.2 |  |  |  |  |  |  |
| Nedstrand et al. (2006) |  |  |  |  |  |  |  |  |  |  |  |  |  |  |  |  |  |  |
| Yang et al. (2021) | EORTC QLQ-C30 | pre: 27.29 ± 10.67 post: 43.75 ± 15.87 | pre: 31.88 ± 10.32 post: 21.44 ± 12.63 |  |  |  | QLQ-C30 | pre: 56.67 ± 14.63 post: 41.39 ± 12.58 | pre: 51.94 ± 19.71 post: 53.13 ± 16.75 | EORTC QLQ-C30 | pre: 45.21 ± 17.49 post: 64.17 ± 16.58 | pre: 44.79 ± 19.31 post: 44.79 ± 19.31 |  |  |  | SDS | pre: 57.85 ± 5.19 post: 37.68 ± 7.62 | pre: 58.15 ± 4.83 post: 49.16 ± 8.53 |
| Zhang et al. (2023) | FACT-B | pre: 80.3 ± 21.3 post: 99.4 ± NA | pre: 82.5 ± 19.7 post: 92.6 ± NA | PSQI | pre: 13.6 ± 3.4 post: 10.2 ± NA | pre: 12.7 ± 3.3 post: 9.4 ± NA | BFI | pre: 5.6 ± 2.1 post: 2.9 ± NA | pre: 5.6 ± 2.1 post: 3.5 ± NA |  |  |  | HADS-A | pre: 9.2 ± 3.9 post: 6.6 ± NA | pre: 8.6 ± 3.2 post: 7.6 ± NA | HADS-D | pre: 8.4 ± 4.1 post: 5.2 ± NA | pre: 8.1 ± 3.8  post: 6.8 ± NA |
| Shin et al. (2016) | PAC-QOL | pre: 1.85 ± 0.549 post: 0.44 ± 0.324 | pre: 2.21 ± 0.809 post: 1.70 ± 0.815 |  |  |  |  |  |  |  |  |  |  |  |  |  |  |  |
| Tang et al. (2014) |  |  |  | PSQI | pre: 9.63 ± 4.72 post: 7.47 ± 4.88 | pre: 9.44 ± 3.76 post: 10.09 ± 4.76 | TFRS | pre: 130.2 ± 62.1 post: 135.1 ± 74.1 | pre: 150.5 ± 63.2 post: 175.7 ± 75.0 |  |  |  | HADS-A | pre: 7.21 ± 3.58 post: 5.07 ± 3.71 | pre: 6.94 ± 3.62 post: 7.18 ± 4.56 | HADS-D | pre: 7.29 ± 4.39 post: 7.93 ± 2.84 | pre: 8.75 ± 3.80 post: 9.73 ± 5.52 |
| Jung et al. (2023) | CIPN-20 | pre: 52.58 ± 14.86 post: 40.16 ± 9.95 | pre: 46.96 ± 11.19 post: 43.14 ± 13.99 |  |  |  |  |  |  |  |  |  |  |  |  |  |  |  |
| Yeh et al. (2016) |  |  |  | VSHSS | pre: 848.76 ± 150.03 post: 945.49 ± 119.50 | pre: 936.76 ± 104.20 post: 590.98 ± 72.70 | QLQ-C30 | pre: 5.20 ± 1.06 post: 0.43 ± 1.42 | pre: 7.45 ± 1.57 post: 5.53 ± 1.71 |  |  |  |  |  |  |  |  |  |
| Zhang et al. (2016) |  |  |  |  |  |  | MFSI-SF | pre: 46.0 ± 11.6 post: 53.3 ± 11.8 | pre: 46.8 ± 12.2 post: 59.3 ± 12.2 | MFSI-SF | pre: 18.4 ± 3.2 post: 19.1 ± 3.9 | pre: 17.5 ± 3.3 post: 19.3 ± 3.5 |  |  |  |  |  |  |
| Zhou et al. (2017) |  |  |  |  |  |  | MFSI-SF | pre: 10.81 ± 7.18 post: 26.40 ± 14.20 | pre: 10.88 ± 7.66 post: 32.36 ± 11.12 | MFSI-SF | pre: 7.47 ± 1.31 post: 9.42 ± 2.66 | pre: 7.50 ± 1.35 post: 10.33 ± 2.16 |  |  |  |  |  |  |
| Chen et al. (2021) | FACT-L | pre: 83.91 ± 6.45 post: 113.29 ± 7.04 | pre: 84.44 ± 6.07 post: 101.73 ± 7.18 |  |  |  |  |  |  |  |  |  |  |  |  |  |  |  |
| Zhang et al. (2024) | QLQ-C30 | pre: 45.2 ± 18.9 post: 69.4 ± 18.3 | pre: 50.5 ± 18.0 post: 51.0 ± 16.6 |  |  |  |  |  |  | QLQ-C30 | pre: 44.1 ± 18.1 post: 73.8 ± 16.4 | pre: 44.6 ± 24.9 post: 55.4 ± 15.8 |  |  |  |  |  |  |
| Zhou et al. (2022) | QLQ-C30 | pre: 48.81 ± 3.38 post: 63.43 ± 6.52 | pre: 49.28 ± 3.69  post: 54.06 ± 4.27 |  |  |  |  |  |  | QLQ-C30 | pre: 49.76 ± 6.02 post: 70.74 ± 5.82 | pre: 50.26 ± 5.52 post: 57.78 ± 4.77 |  |  |  |  |  |  |
| Zhang et al. (2020a) | Karnofsky | pre: 66.98 ± 9.11 post: 68.10 ± 7.21 | pre: 67.02 ± 8.32 post: 60.88 ± 7.65 |  |  |  |  |  |  |  |  |  |  |  |  |  |  |  |
| Zhang et al. (2020b) | Karnofsky | pre: 85.0 ± 5.0 post: 85.5 ± 5.5 | pre: 85.0 ± 5.0 post: 76.0 ± 6.0 |  |  |  |  |  |  |  |  |  |  |  |  |  |  |  |
| Peng et al. (2016) | FACT-H&N量 | 87.39 ± 13.791 | 72.88 ± 8.749 |  |  |  |  |  |  | FACT-H&N量 | 16.70 ± 2.698 | 13.24 ± 2.016 |  |  |  |  |  |  |
| Zhou et al. (2024) | EORTC QLQ-C30 | pre: 65.00 ± 8.01 post: 69.17 ± 6.62 | pre: 63.89 ± 5.93 post: 59.72 ± 4.93 |  |  |  |  |  |  | EORTC QLQ-C30 | pre: 82.22 ± 10.43 post: 86.94 ± 6.81 | pre: 80.83 ± 7.94 post: 76.39 ± 7.61 |  |  |  |  |  |  |
| Wu et al. (2022) | QLQ-C30 | 75.38 ± 3.49 | 72.46 ± 3.51 | Sleep Disorders Diagnostic Scale | 2.41 ± 0.78 | 3.47 ± 0.84 |  |  |  |  |  |  |  | pre: 60.28 ± 4.97 post: 46.36 ± 3.63 | pre: 60.07 ± 4.86 post: 49.76 ± 3.87 |  | pre: 60.67 ± 4.86 post: 47.61 ± 3.79 | pre: 60.12 ± 4.67 post: 50.35 ± 3.97 |
| Wang et al. (2020) | QLQ-C30 | pre: 83.05 ± 5.54 post: 68.03 ± 4.22 | pre: 83.45 ± 5.47 post: 74.58 ± 5.21 | PSQI | pre: 1.92 ± 0.49 post: 0.95 ± 0.37 | pre: 1.97 ± 0.44 post: 1.32 ± 0.42 |  |  |  | FACT-L | pre: 16.83 ± 3.16 post: 9.75 ± 2.15 | pre: 16.54 ± 3.19 post: 12.54 ± 2.66 |  |  |  |  |  |  |
| Xiao et al. (2022) | QLQ-C30 | 46.13 ± 2.15 | 33.09 ± 2.73 | PSQI | pre: 12.58 ± 2.42 post: 7.06 ± 1.56 | pre: 14.95 ± 2.55 post: 11.75 ± 2.07 |  |  |  |  |  |  |  |  |  |  |  |  |

# Supplementary 7: Publication bias


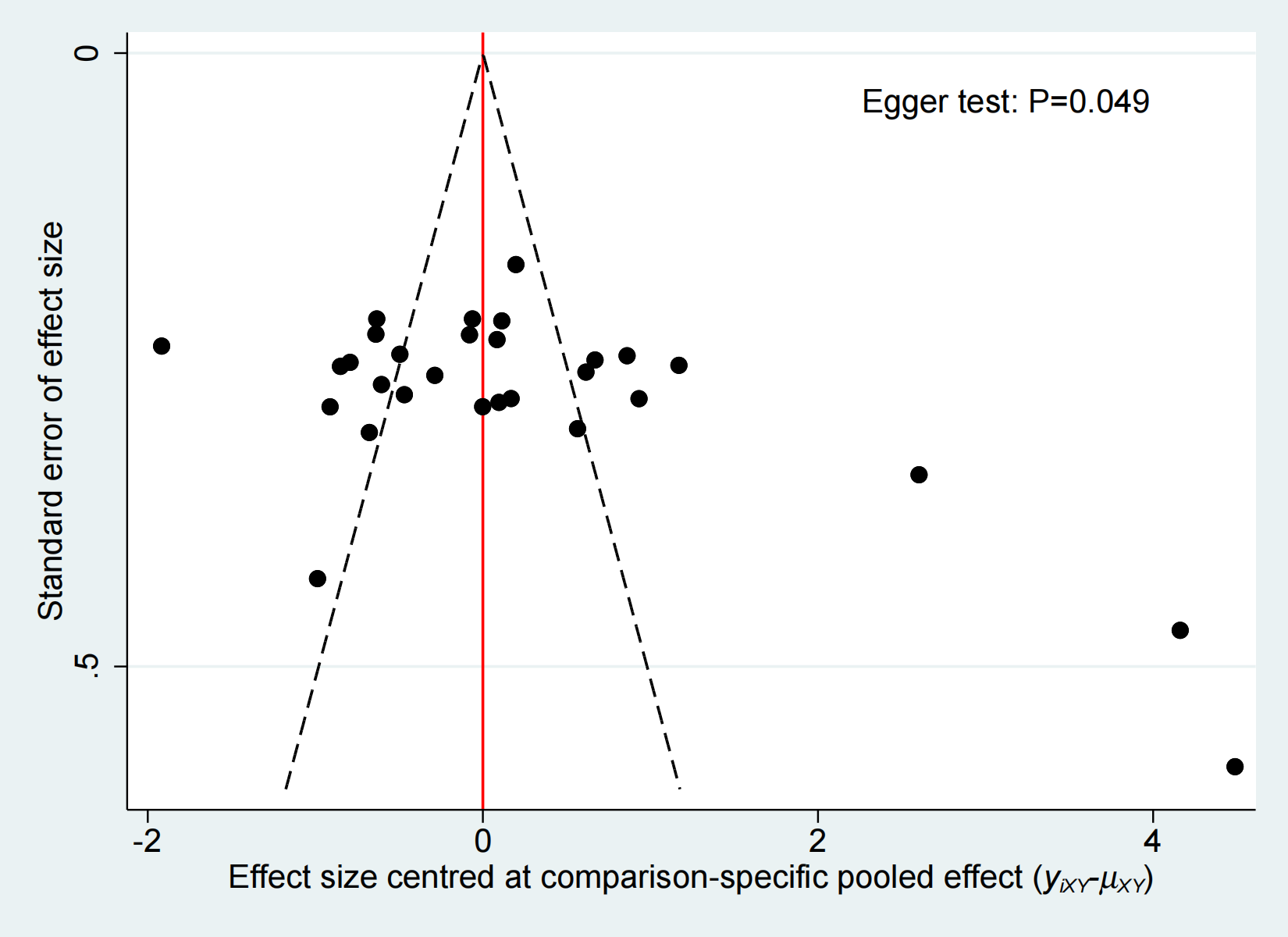


Figure 7.1 The funnel plot of QoL. The result of Egger test showed the p=0.049.


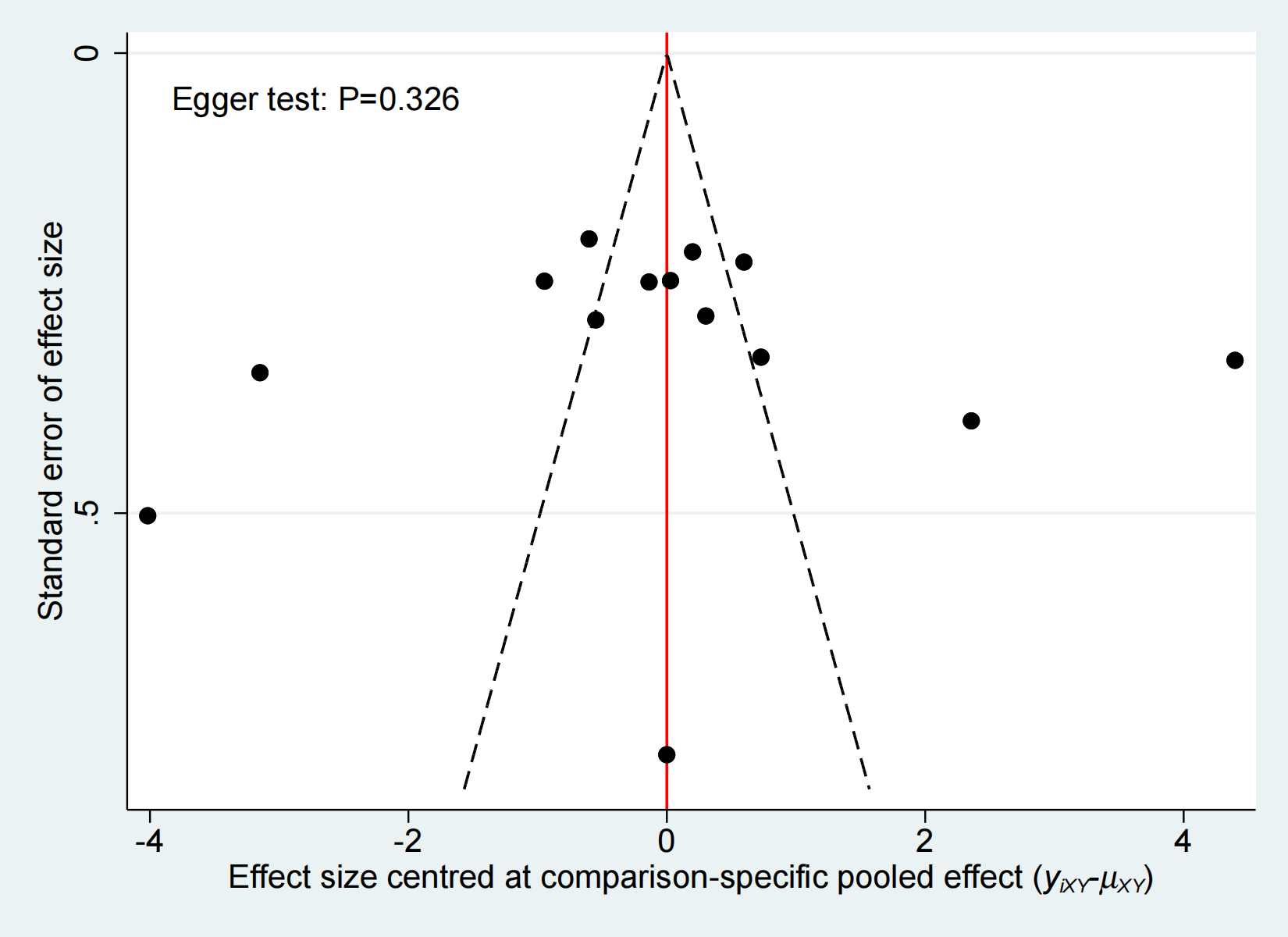


Figure 7.2 The funnel plot of sleep quality. The result of Egger test showed the p=0.326.


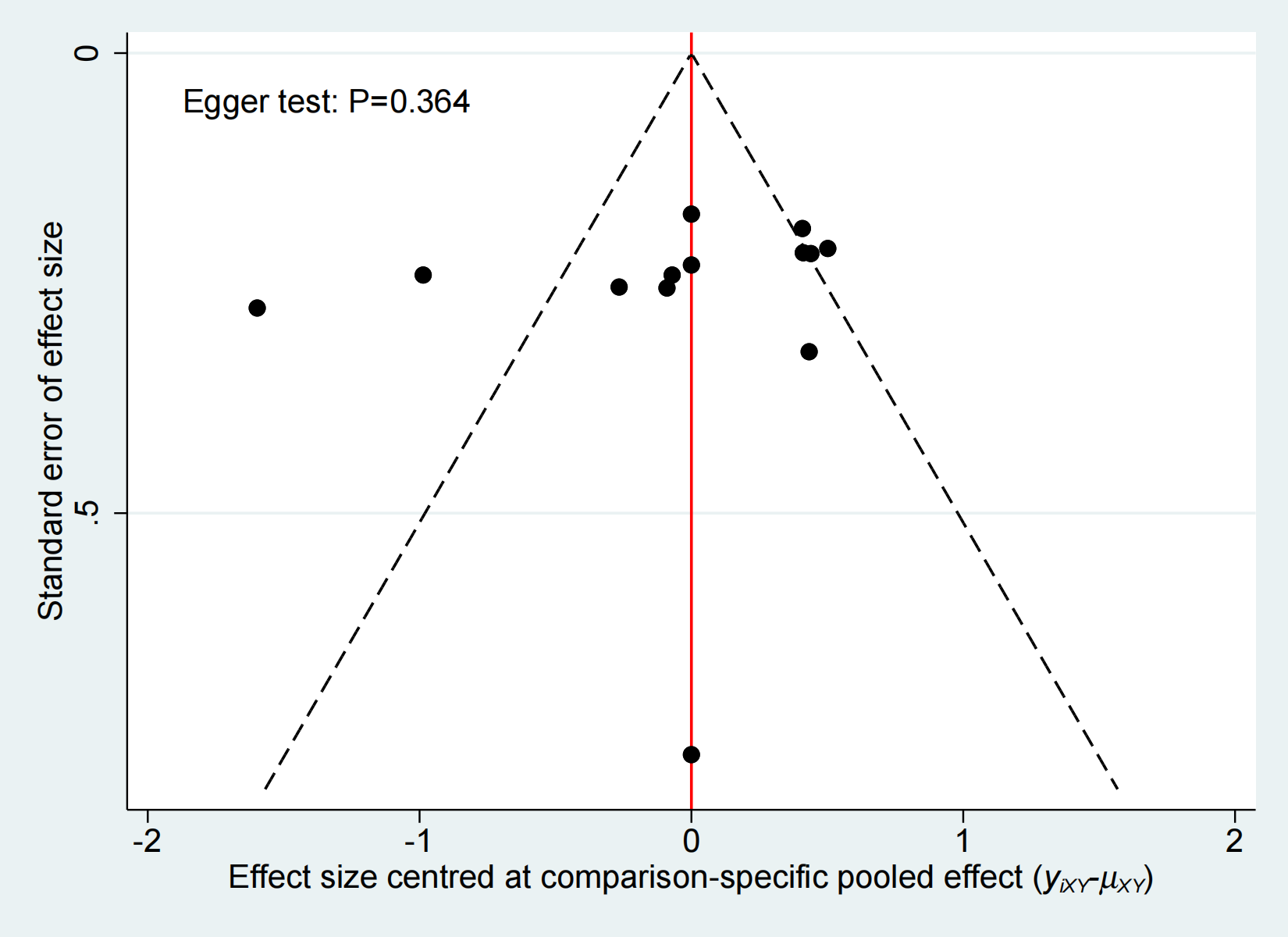


Figure 7.3 The funnel plot of fatigue. The result of Egger test showed the p=0.364.


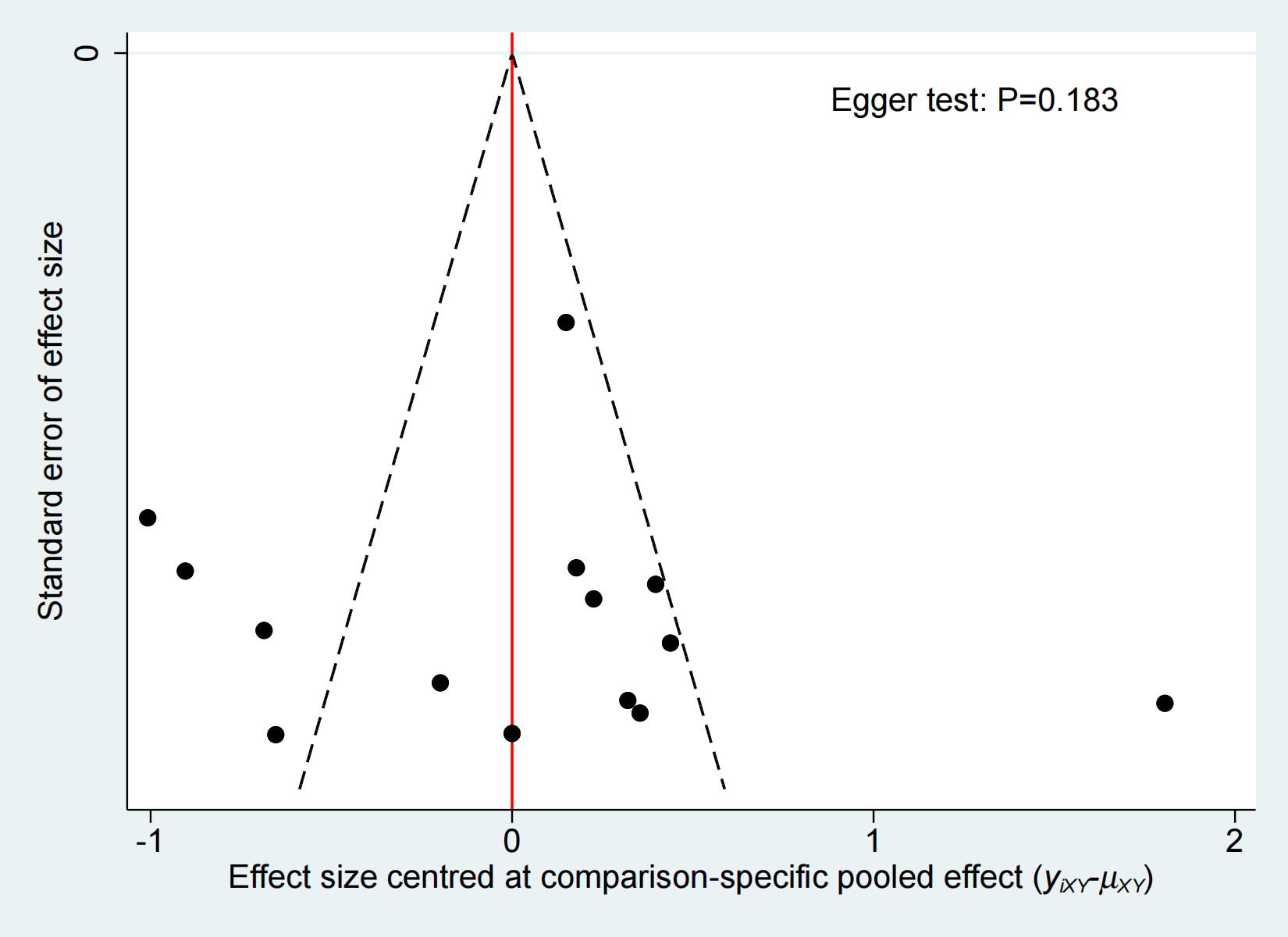


Figure 7.4 The funnel plot of general mood. The result of Egger test showed the p=0.183.


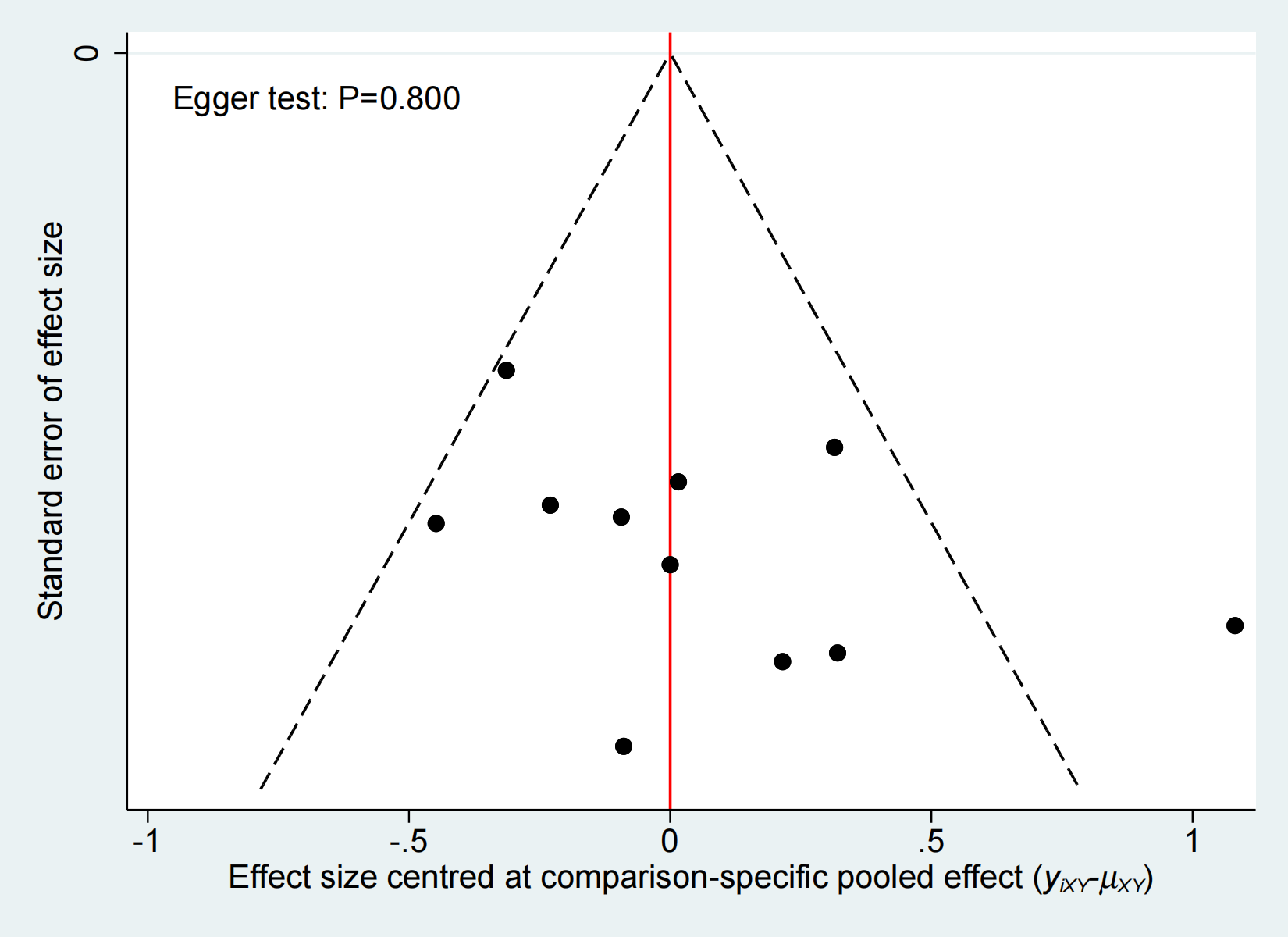


Figure 7.5 The funnel plot of anxiety. The result of Egger test showed the p=0.800.


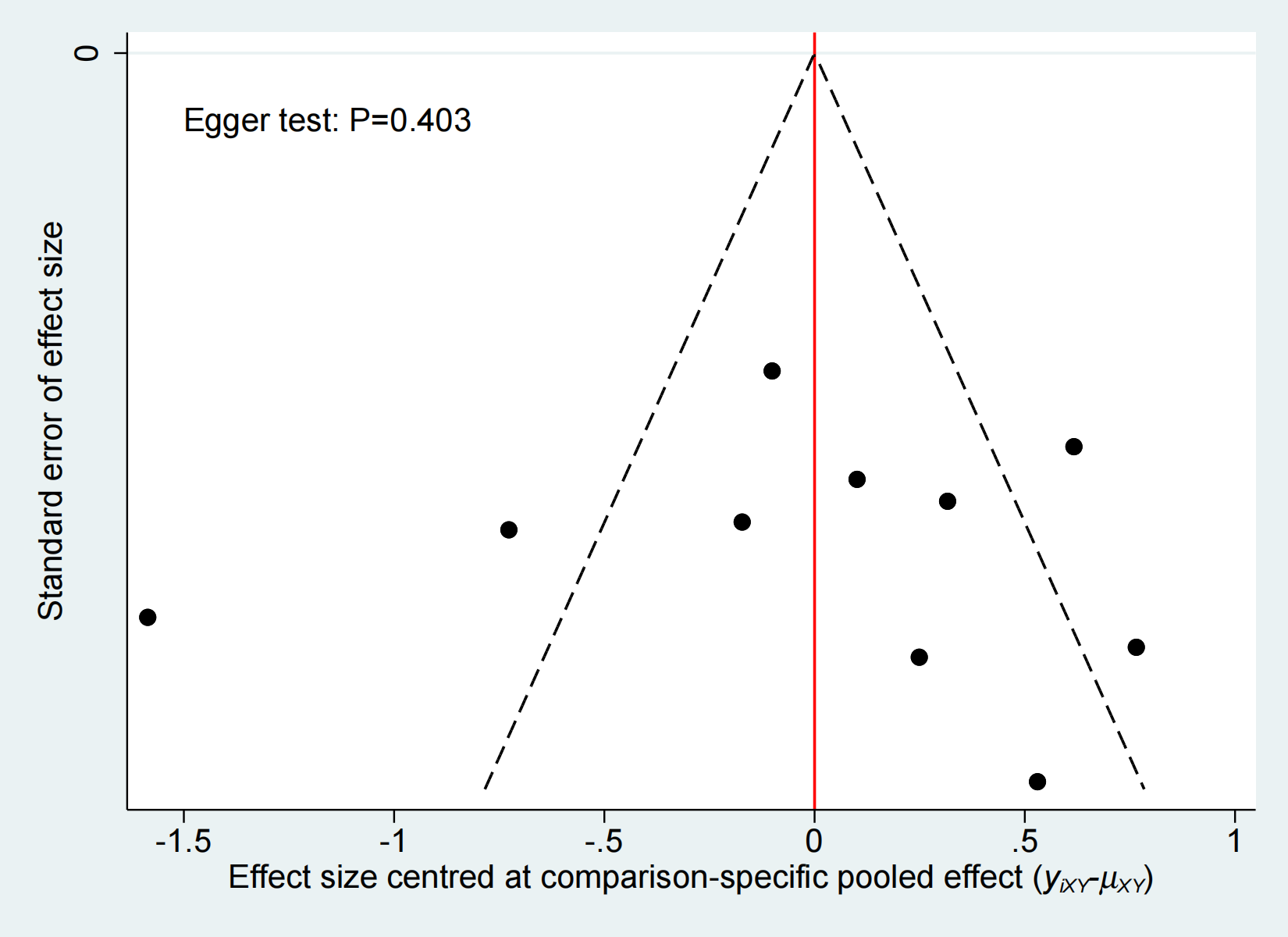


Figure 7.6 The funnel plot of depression. The result of Egger test showed the p=0.403.
